# A fresh look to the phenotype in mono-allelic likely pathogenic variants of the leptin and the leptin receptor gene

**DOI:** 10.1186/s40348-021-00119-7

**Published:** 2021-08-26

**Authors:** Ingrid Koerber-Rosso, Stephanie Brandt, Julia von Schnurbein, Pamela Fischer-Posovszky, Josef Hoegel, Hannah Rabenstein, Reiner Siebert, Martin Wabitsch

**Affiliations:** 1 Division of Pediatric Endocrinology and Diabetes, Department of Pediatrics and Adolescent Medicine, University Medical Center Ulm, Ulm, Germany; 2 Institute of Human Genetics, University of Ulm, University Medical Center Ulm, Ulm, Germany

**Keywords:** LEP, LEPR, Phenotype, Animal, Humans

## Abstract

Leptin (LEP) and leptin receptor (LEPR) play a major role in energy homeostasis, metabolism, and reproductive function. While effects of biallelic likely pathogenic variants (*-/-*) on the phenotype are well characterized, effects of mono-allelic likely pathogenic variants (*wt/-*) in the *LEP* and *LEPR* gene on the phenotype compared to wild-type homozygosity (*wt/wt*) have not been systematically investigated. We identified in our systematic review 44 animal studies (15 on *Lep*, 29 on *Lepr*) and 39 studies in humans reporting on 130 mono-allelic likely pathogenic variant carriers with 20 distinct *LEP* variants and 108 heterozygous mono-allelic likely pathogenic variant carriers with 35 distinct *LEPR* variants. We found indications for a higher weight status in carriers of mono-allelic likely pathogenic variant in the leptin and in the leptin receptor gene compared to *wt/wt*, in both animal and human studies. In addition, animal studies showed higher body fat percentage in *Lep* and *Lepr wt/-* vs *wt/wt*. Animal studies provided indications for lower leptin levels in *Lep wt/-* vs. *wt/wt* and indications for higher leptin levels in *Lepr wt/-* vs *wt/wt*. Data on leptin levels in human studies was limited. Evidence for an impaired metabolism in mono-allelic likely pathogenic variants of the leptin and in leptin receptor gene was not conclusive (animal and human studies). Mono-allelic likely pathogenic variants in the leptin and in leptin receptor gene have phenotypic effects disposing to increased body weight and fat accumulation.

**Supplementary Information:**

The online version contains supplementary material available at 10.1186/s40348-021-00119-7.

## Introduction

More than 20 years of research reveals the fundamental role of leptin (LEP) and its receptor (LEPR) in multiple pathways involved in body weight regulation and energy homeostasis, as well as in growth, fertility, and immune function [1–3]. The occurring of a biallelic likely pathogenic variant (*-/-*) leading to obesity and infertility in mice was first described by Ingalls et al. who named the responsible gene the obese “*ob*” gene [4]. Later, the C to T variant changing an arginine codon to a stop codon at position 105 in the *ob* gene was detected and the product of *ob* was identified in the adipose tissue. Its role as a central component in a feedback signaling on stored energy amount to the hypothalamus was discussed [5]. This protein was later described as *leptin*, derived from the Greek root *leptos*, meaning thin [6]. An additional biallelic likely pathogenic variant leading to obesity regardless of diet restriction was observed in mice, which additionally showed hyperglycemia and infertility. The authors named the causative gene diabetes “*db*” [7]. It was later proven that the *db* mutation consists in a G to T transversion leading to abnormal splicing of the leptin receptor [8, 9]. The fatty gene *fa*, responsible for obesity in -/- rats, was shown to be orthologous to the *db* gene [10]. Through parabiosis experiments, Coleman et al. showed that *ob* mice lacked a certain “satiety factor”, while in *db* mice, a receptor in the “satiety centers” sensitive to the satiety factor was missing [11, 12].

Leptin (LEP) is a cytokine mainly produced in the adipose tissue which activates the leptin receptor (LEPR), predominantly expressed in the central nervous system (CNS) in the arcuate nucleus [13, 14]. Leptin and its receptor govern energy intake and expenditure primarily through regulation of neuropeptide synthesis and secretion in the CNS. In addition, leptin’s peripheral actions have been observed, amongst other tissues, in the skeletal muscle, in the intestine, and in the liver [15–18]. After the first reports on humans with early-onset severe obesity carrying biallelic likely pathogenic variants in the leptin (*LEP)* [19] and leptin receptor gene (*LEPR)* [20], it was clear that a fundamental failure in LEP and LEPR signaling causes a dramatic phenotype. Monogenic obesity associated with *LEP*/*LEPR* variants is inherited through an autosomal recessive pattern [21, 22]. Cardinal symptoms of congenital leptin deficiency due to *LEP* -/- variants include severe early-onset obesity, hyperphagia, and disturbance gonadotropic function [23, 24]. Homozygous loss-of-function variants in *LEPR* also lead to severe early-onset obesity, hyperphagia, and hypogonadotropic hypogonadism [25]. The prevalence of mono-allelic likely pathogenic variants (*wt/-*) of potentially damaging variants in *LEP* has been estimated to amount about 1:1000 [26]. A prevalence of loss-of-function *LEPR wt/-* mutation of about 7:1000 can be assumed [27]. In general, slight phenotypic effects of *wt/-* in autosomal recessive diseases cannot be excluded [28]. Mono-allelic likely pathogenic variants in proteins with structural similarity to LEP/LEPR are known, comparable to variants in growth hormone [29], growth hormone receptor [30], and G-CSF receptor [31]. In one study of Farooqi et al., *LEP wt/-* showed lower leptin levels and increased prevalence of obesity than controls [32].

We hypothesized effects of mono-allelic likely pathogenic variants in the *LEP/LEPR* gene on weight status, body fat percentage, leptin levels, and metabolic risk factors in animal and humans. We performed a systematic review exploring reported animal and human phenotypes in *LEP/LEPR* mono-allelic likely pathogenic variants. We present observed results focusing on weight status, body fat, leptin levels, and metabolic features in *Lep/Lepr* animal model and in human subjects carrying rare *LEP/LEPR* mono-allelic likely pathogenic variants, particularly in comparison to the phenotype in the wildtype homozygosity condition.

## Methods

### Literature research

We performed a systematic literature review on animal and human studies that reported the phenotype in mono-allelic likely pathogenic variants for the *LEP* or *LEPR* gene (*wt/-*). The literature research was performed in PubMed NCBI. Identified abstracts were screened and according to defined inclusion and exclusion criteria, full-text articles were sought and then reviewed by two independent reviewers. Eligibility criteria were full-text availability in English language and published status.

Search criteria used for systematic literature review on the phenotype in mono-allelic likely pathogenic variants in the *LEP* or *LEPR* gene in the animal model were: “heterozygous leptin mice,” “heterozygous leptin receptor mice,” “heterozygous leptin animals,” “heterozygous leptin receptor animals,” “fa heterozyg*,” “ob heterozyg*,” and “db heterozyg*,” (date last searched March 1, 2021). We included articles reporting the phenotype of mono-allelic likely pathogenic variants in the *Lep/Lepr* gene in animal models as well as of *wt/wt* animals. Exclusion criteria were reviews not presenting original data, intervention studies without baseline phenotype, studies using *Lep/Lepr wt/-* animals as models for studies on tumorigenesis, gestational diabetes or development of other diseases without information on baseline phenotype, double mutants, or animals with combined mutation status. In animal studies, baseline data on body mass, body weight, body fat, leptin levels, metabolic parameters, hyperphagia, immunological phenotype, and puberty development were extracted.

For the systematic literature review on the phenotype in mono-allelic likely pathogenic variants in the in the *LEP/LEPR* gene in humans, used search criteria were “leptin heterozyg* human,” “leptin receptor heterozyg* human,” “rare variant LEP,” and “rare variant LEPR” (date last searched March 1, 2021). We included articles expressively reporting humans with rare mono-allelic likely pathogenic variants in the *LEP* or in *LEPR* gene. Exclusion criteria were articles only reporting polymorphisms (minor allele frequency > 0.01), low frequency variants, compound or double heterozygotes, reviews not presenting original data, publications without information on BMI or weight status, and publications whose full text was not available in English language. Extracted data consisted of information on age, sex, birth weight, weight status, fat mass, leptin levels, comorbidities, and metabolic parameters. Moreover, information on hyperphagia, puberty as well as reproductive status, and immunological status was searched. In humans, data was extracted for mono-allelic likely pathogenic variants (*wt/-*), wild- type homozygosity (*wt/wt*), and biallelic likely pathogenic variants group (*-/-*). Data in *-/-* relatives was extracted to better evaluate the phenotype of mono-allelic likely pathogenic variants within described families and to better correlate the phenotype of mono-allelic likely pathogenic variants with the spectrum of different variants reported. Subjects aged 0–19 years were classified as children, and subjects older than 19 years or parents were classified as adults. Four siblings (*n* = 3 *LEP wt/wt* siblings, one *LEP wt/-* sibling) in the study of Montague et al. [19] with no reported age were classified as children. Three *LEP* wt/- subjects were classified as adults based on information in the text [33].

In the description of the results and in the discussion of the findings the term “heterozygous” (wt/-) is used instead of “mono-allelic likely pathogenic variant,” the term “homozygous” (-/-) is used instead of “biallelic likely pathogenic variant,” and the term “wild-type” (wt/wt) is used instead of “wild-type homozygosity” for better readability.

### Weight status and BMI z score calculation

In human subjects, we evaluated the weight status based on qualitative reporting and on available anthropometric data for BMI *z* score calculation. When human data on sex, age, weight, height, or BMI were available, we calculated BMI *z* scores according to WHO criteria [34] to obtain comparable values. For calculation of the BMI *z* score in subjects older than 19 years, an age of 19 years was assumed. The normal weight was defined as BMI *z* score − 2 to 1, overweight was defined as BMI *z* score > 1, obesity as BMI *z* score > 2, and thinness as a BMI *z* score < -2.

### Leptin serum concentrations

To describe leptin levels in the human *LEP*/*LEPR wt/-*, *-/-*, and in the *wt/wt* group, we extracted values reported in literature and calculated mean and range values. In the case of *LEP* variants leading to bioinactive leptin (p.D100Y and p.N103K), we included only bioactive leptin levels for calculations of mean leptin and for plotting in Fig. 4A. Bioactive leptin levels measured with the reported assay are highly correlated to immunoreactive leptin levels measured in patients showing leptin variants which do not lead to bioinactivity or in healthy controls [35], so that leptin concentrations could be compared in Fig. 4A. For calculations of mean leptin levels in *LEPR wt/-* and plotting in Fig. 4B, values from the study of Clement [20] have not been included, since the reported *LEPR* variant causes a truncated receptor which binds to serum leptin and thus to apparently elevated blood leptin values. The same *LEPR* variant is described in two further studies, which also report very high leptin levels due to truncated receptor binding. In these studies, also free (not-bound) leptin levels in variant carriers were reported [36, 37]. Thus, these free leptin levels were used for calculations of mean leptin levels and for plotting in Fig. 4B.

### Body fat percentage

We extracted information on body fat percentage from 5 studies on human *LEP wt/-* subjects and 4 studies on human *LEPR wt/-* subjects. Methods of body fat measurement varied in the different studies (biphotonic absorptiometry, electric impedance, or dual energy X-ray absorptiometry scanning reported), and described values are reported in Table S2, 3 (Table S2, 3, Mean body fat%). For values in the study of Karvonen et al. 1998 [38], in which fat mass in kilograms and body weight in kilograms were reported, we calculated body fat percentage and reported it in Table S2.

### Evaluation of reported data on the phenotype

A Animal studies: Three authors (Koerber-Rosso, Brandt, and Wabitsch) evaluated the reported data on weight (W), leptin levels (L), and metabolic parameters (M) separately for *Lep wt/-* (Table S1a) and *Lepr wt/-* (Table S1b) compared to *wt/wt* animals, respectively, for each of the published studies. Results were reported as follows:
  1. W + , if there was a significant difference in body weight and/or body fat mass reported, otherwise as W-
  2. L + , if there was a significant difference for circulating leptin levels reported, otherwise as L-
  3. M + , if there was a significant difference in metabolic parameters reported, otherwise as M-.
B Human studies: Three authors (Koerber-Rosso, Brandt, and Wabitsch) evaluated the reported data on weight (W), circulating leptin levels (L), and metabolic parameters (M) separately for humans with *LEP wt/-* (Table S2) and *LEPR wt/-* (Table S3) compared to *wt/wt*, respectively, for each of the published studies. Results were reported as follows:
  1. W + , if the BMI *z* score was > 1 in one group (*LEP wt/-* or *wt/wt*) and not in the other, or if the reported BMI range was not overlapping between *wt/-* individuals and *wt/wt* and/or if the reported range of body fat % was not overlapping between *wt/-* individuals and *wt/wt*, otherwise as W-
  2. L + if the reported range of leptin levels was not overlapping between *wt/-* individuals and *wt/wt*, otherwise as L-
  3. M + if a significant difference in metabolic parameters was reported between heterozygous individuals and WT in the individual study, otherwise as M-.

### Human *LEP/LEPR* variants and possible pathogenic effects

In Table S2 and 3 (Variant/c.DNA/p.position), the amino acid position in the immature leptin protein is reported as described in respective studies. Wherever possible, we used the nomenclature adhering to the recommendations of the Human Genome Variation Society [39]. Possible pathogenic effects of reported variants are listed as described in cited studies. Mostly, variants were described in mutated homozygosity carriers, so that high penetrance and pathogenicity can be assumed. If no assessment of pathogenicity was presented, we assessed pathogenicity by using the prediction tool PolyPhen-2 and SIFT (Table S2, 3: Possible pathogenic consequence). Transcripts used for PolyPhen-2 analyses were transcript ENST00000308868.5 for leptin (ENSG00000174697, NM_000230.3, P41159) and transcript ENST00000349533.10 for leptin receptor (ENSG00000116678, NM_002303.5, P48357). Many reviewed studies did not report transcript number, but it can be assumed that they referred to the above reported transcripts, since the aminoacid position was corresponding.

### Statistical analysis

Statistical analyses were computed using SAS 9.2 (SAS 9.2, SAS Institute Inc., Cary, North Carolina). Data are presented as mean and range. Differences in BMI *z* score values, in body fat (%), and in leptin levels between *wt/wt*, *wt/-*, and *-/-* carriers of a *LEP* or *LEPR* variant in children as well as in adults were tested by using the Kruskal–Wallis test (non-parametric). If the *p* value of the Kruskal–Wallis test was lower than 0.05, we used the Mann–Whitney *U* test to identify differences between two groups. A *p* value (two-sided) of less than 0.05 was considered as statistically significant. Differences in BMI *z* score, in BF% as well as in leptin levels between *wt/wt*, *wt/-*, and *-/-* carriers of a *LEP* or *LEPR* variant in children as well as in adults are presented as box plots (including individual data). Graphs were computed using Graph Pad Prism 7 (Graph Pad Software Inc., San Diego, CA).

## Results

### Comparison of phenotype between *Lep wt/*- and *wt/wt* (animals)

We identified 15 studies describing the phenotype in *Lep wt/-* compared to *wt/wt* animals (Table S1 A). Fourteen studies were performed in mice [18, 40–52], and one study was performed in cows [53].

*Weight status*: Fourteen studies reported weight status in *wt/-* and *wt/wt* mice. In seven studies, no difference between *Lep wt/-* and *wt/wt* was observed [18, 42, 43, 45, 46, 51, 52]. In six studies, significantly higher weight status for *Lep wt/-* and *wt/wt* was observed [40, 41, 44, 47, 49, 50], although two of these studies only investigated female mice [47, 50] and one only male mice [40]. In one out of the six studies, significantly higher weight status was observed only in female and not in male *Lep wt/-* mice [44], and in another study only in male *Lep wt/-* mice [41]. In the one study on cows, significantly lower body weight in *Lep wt/-* vs *wt/wt* was reported [53].

*Body fat*: The body fat content in *Lep wt/-* mice was reported in three studies and all reporting significantly higher body fat than in WT mice [40, 41, 51]. In a further study, significantly higher white adipose tissue weight was found in *Lep wt/-* vs WT [47].

*Leptin levels*: Five studies reported leptin levels in *Lep wt/-* compared to *wt/wt* mice. Three of them observed that leptin levels were similar in *Lep wt/-* and *wt/wt*. But in relation to body fat, they were significantly lower in *LEP wt/-* compared to *wt/wt* [40, 41, 51]. Two studies observed significantly lower mean leptin levels than in *wt/wt* without relating them to body fat [47, 49].

*Metabolic parameters*: Metabolic parameters in *Lep wt/-* and *wt/wt* were described in 10 studies. In four of them, varying differences were observed under standard conditions, including significantly higher glucose and insulin levels [43]; significantly higher fasting blood glucose in female *Lep wt/-* mice [44]; significantly higher triglyceride levels but normal insulin, glucose, and cholesterol [50]; and slight glucose intolerance and significantly higher alanine aminotransferase [40]. In further four studies, no differences in metabolic parameters including blood glucose and insulin concentrations were observed [18, 42, 46, 52]. Interestingly, in studies investigating metabolic differences occurring under high-fat diets, greater metabolic impairment was observed in *Lep wt/-* vs *wt/wt* mice, comprising greater loss of glucose tolerance, hypercholesterinemia, and hepatic steatosis [40] higher insulin levels in male mice [51] and higher serum cholesterol and glucose [49].

*Eating behavior*: Two of the reviewed studies commented on eating behavior and observed that *Lep wt/-* consumed more food compared to *wt/wt* mice [43, 47].

*Pubertal development, immune system*: No animal study investigating the effect of the *Lep wt/-* genotype vs *wt/wt* on pubertal development or on the immune system was found.

### Comparison of phenotype between *Lepr wt/*- and *wt/wt* (animals)

A total of 29 animal studies reporting the phenotype in *Lepr wt/-* and *wt/wt* animals were evaluated (Table S1B). Fourteen studies were performed in mice [41, 42, 54–65], fourteen studies in rats [10, 66–78], and one study in sheep [79].

*Weight status*: 28 studies provided information on body weight in *Lepr wt/-* and *wt/wt*. Out of these studies, 11 observed no differences in weight status between *Lepr wt/-* and *wt/wt* [41, 42, 54, 56, 69, 70, 72, 73, 75–77]. Eight studies observed significantly higher body weight in *Lepr wt/-* [10, 55, 57, 65–67, 78], including one study reporting higher weight in *Lepr wt/-* sheep vs *wt/wt* only at adult age [79]. Four further studies observed significantly lower body weight in *Lepr wt/-* animals than in *wt/wt* [58, 62, 71, 74]. Several studies investigated body weight in pregnant *Lepr wt/-* mice vs *wt/wt* mice. Three studies reported significantly higher weight in pregnancy in *Lepr wt/-* than in *wt/wt* dams [61, 63, 65], one study reported additionally higher prepregnant body weight in *Lepr wt/-* than *wt/wt* female mice [60]. Lower weight of offspring of *Lepr wt/-* mothers was observed in one study [61], whereby higher weight in offspring of *Lepr wt/-* [61, 63], or similar weight in offspring of *Lepr wt/-* and *wt/wt* dams were also reported [65].

*Body fat*: Information on fat mass or weight of fat pads both in heterozygous *Lepr wt/-* and *wt/wt* animals was provided in 16 studies. Most studies (*n* = 12) observed significantly higher fat mass or higher weight of fat pads in *wt/-* animals [10, 41, 56, 60, 64–68, 74, 75, 78]. One study observed significantly lower fat mass in *Lepr wt/-*, but considering lower body weight found in *Lepr wt/-* than in *wt/wt*, in total also here higher body fat percentage than in *wt/wt* was observed [71]. Three studies found no difference in fat mass in *Lepr wt/-* animals compared to *wt/wt*. Among those, one of them investigated only brown adipose tissue [77], the other two reported retroperitoneal and intrascapular fat pad’s [72] and epidydimal fat pad’s [69] weight, respectively.

*Leptin levels*: We found information on leptin levels in *Lepr wt/-* and *wt/wt* in animals in 11 studies. Five studies described significantly higher blood leptin levels for *Lepr wt/-* compared to *wt/wt* [41, 64, 67, 69, 78]: in two of these, leptin levels were found to be higher in in *Lepr wt/-* than in *wt/wt* also if related to fat mass [41, 78]. One study reported significantly higher leptin levels in homogenized adipose tissue of *Lepr wt/-* than in *wt/wt* [68]. Moreover, in three articles [59–61], hyperleptinemia during pregnancy of *Lepr wt/-* mice was observed. On the other hand, two studies [56, 72] observed comparable leptin levels between *Lepr wt/-* and *wt/wt*.

*Metabolic parameters*: Information on metabolic parameters in *Lepr wt/-* and *wt/wt* animals was provided in 24 studies. In most studies (*n* = 15) [42, 54–56, 64, 66–68, 72, 73, 75, 76, 78], including two studies on pregnant *Lepr wt/-* mice [60, 61], no differences between *Lepr wt/-* and *wt/wt* were observed in provided metabolic parameters. Abnormal metabolic values in comparison to *wt/wt* were observed in *Lepr wt/-* in eight studies [57, 62, 69, 74, 77], three of which reported status in pregnant *Lepr wt/-* mice [59, 63, 65]. Abnormalities in *Lepr wt/-* included significantly higher insulin levels [57, 65, 69], higher fasting blood glucose [57, 62, 65], higher cholesterol [74], higher triglycerides [57, 69, 74], and higher fatty acid levels [77]. In one study, *Lepr wt/-* mice showed lower blood glucose than *wt/wt* [58].

*Pubertal development, immune system*: An investigation on sheep showed that *LEPR wt/-* sheep had a significantly higher risk to fail to undergo puberty before 1 year of age compared to *wt/wt* [79]. No other studies investigating puberty onset in *Lepr wt/-* animals were found. No study investigated the effect of the *Lepr wt/-* vs *wt/wt* genotype on the immune system.

*Eating behavior:* Eating behavior was reported in five studies. Four did not observe hyperphagia in *Lepr wt/-* mice compared to *wt/wt* [69, 72], two of them investigating pre-pregnant state [64, 65], while higher eating amount than in *wt/wt* was observed in two studies [70], one of them performed in pregnant mice [65].

### Phenotype in human carriers of *LEP wt/*- variants

**Fig. 1:**
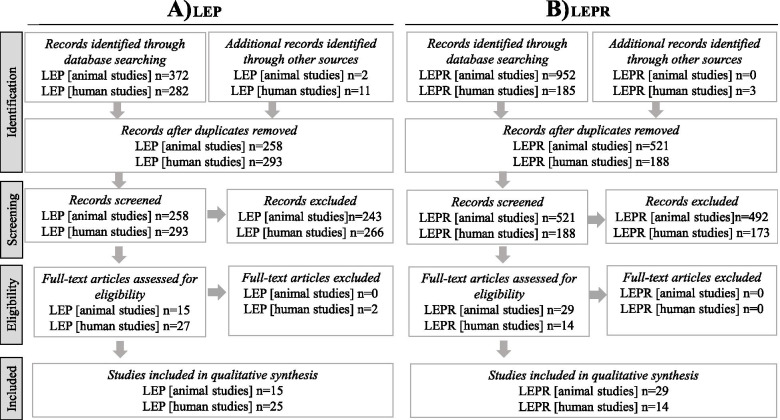
Flowchart of the included studies reporting phenotype in animals as well as in humans with mono-allelic likely pathogenic variant **A** in the *LEP* and **B** in the *LEPR* gene

We reviewed 25 studies reporting on 130 carriers of rare *wt/- LEP* variants (114 adults, children, 7 subjects with no reported age), showing 20 different *LEP* variants (Fig. 1C, D and Table S2). Among the 130 carriers, 61 were male subjects and 51 female subjects, whereas in further 18 individuals, sex category was not reported. Out of 114 adults, 41 were reported to be normal weight, 23 as overweight, and 28 as obese, while the weight status was not reported in the remaining in 22 subjects. Out of the nine children, seven were normal weight, one was overweight, and one showed thinness. Out of the subjects with not reported age, three were normal weight, whereby no information was provided for the other four.

*Weight status in LEP heterozygotes compared to WT:* In eight studies, information on the weight status in *LEP wt/-* as well as in *wt/wt* relatives [19, 32, 33, 37, 80–83] was provided (Table S2). We found slight differences in regard to weight status between *LEP wt/-* variant carriers and *wt/wt* subjects in seven out of eight studies comparing *wt/-* with *wt/wt* relatives. In six of them, a slightly increased frequency of overweight or obesity was observed in adults with *LEP wt/-* vs *wt/wt* [19, 32, 33, 37, 82, 83]. In contrast, the related children with *LEP wt/-* reported in two studies [19, 82] showed a normal weight. The subjects showing overweight or obesity carried heterozygous *LEP* variants p.G133Vfs*15, p.R105W, p.N103K, and p.C117F. A leptin secretion defect has been shown in functional studies for p.G133Vfs*15 and p.R105W and had been predicted for p.C117F. Variant p.N103K leads to leptin bioinactivity [84]. On the other side, in one study [81], out of the seven showing differences in weight status between *LEP wt/-* variants and *wt/wt*, underweight and low body weight were found in two carriers of the heterozygous *LEP* p.P23R variant, putatively leading to increased leptin activity (Table S2); however, the third relative carrying this variant had normal weight. In the last one out of the eight studies comparing *LEP wt/-* to *wt/wt*, *LEP* heterozygotes, as well as *wt/wt* relatives were normal weight [80].

*Weight status in LEP heterozygotes compared to non-related control group*: In six studies, information on *LEP* heterozygotes as well as on non-related control groups was provided. Compared to control groups, *LEP wt/-* showed normal weight and no differences in weight status in two out of the six studies [85, 86] (Table S2). In a further study, *LEP wt/-* showed normal weight, while the control group was BMI-matched to *-/-* subjects. In the other three studies describing subjects with heterozygous variants p.R105W and p.G133Vfs*15, increased frequency of obesity in *wt/-* subjects than in control groups was observed [32, 87, 88].

**Fig. 2:**
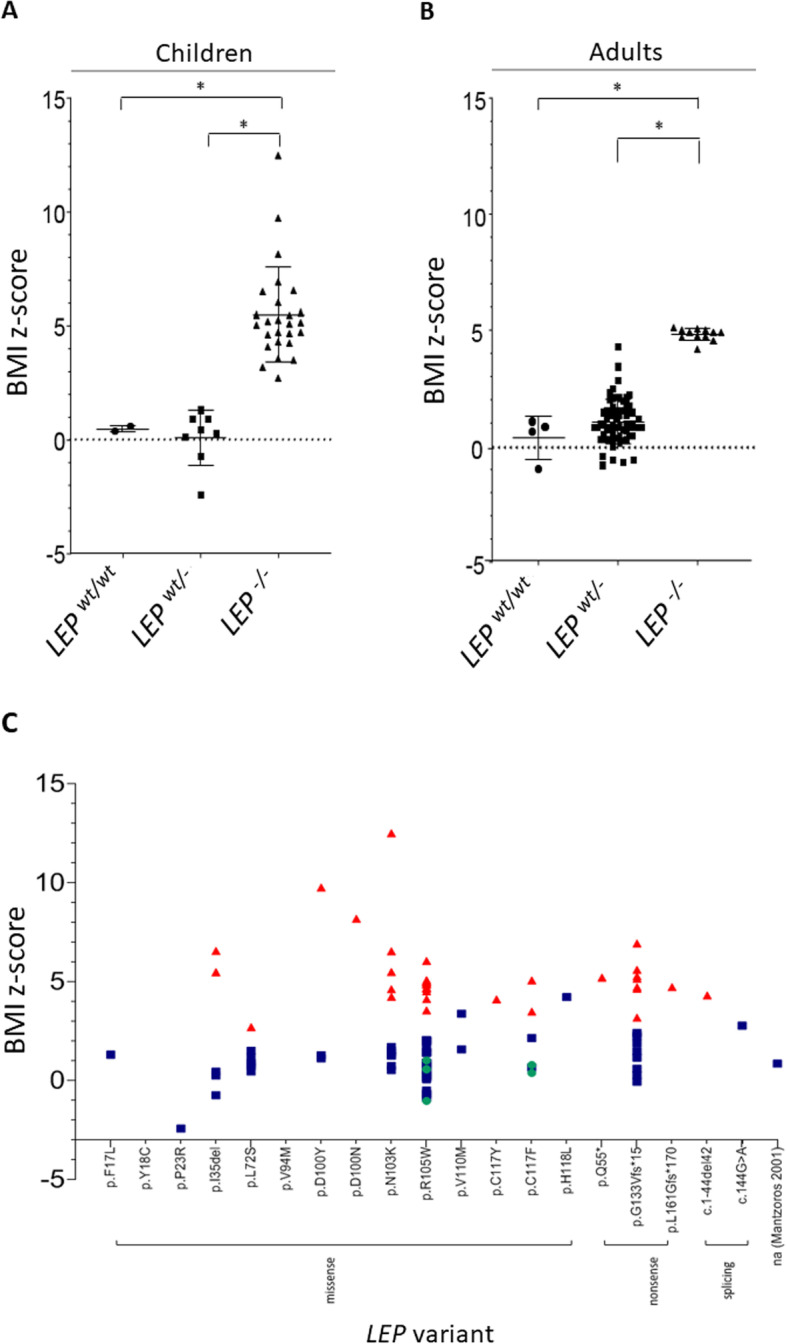
**A, B** Illustration of individual BMI *z* scores in **A** children and **B** adults depending on *LEP* genotype (*wt/wt* vs. *wt/-* vs. -/-). **A** Children: *LEP wt/wt* (*n* = 2): mean: 0.5 (range 0.4–0.6); *LEP wt/-* (*n* = 8): mean − 0.1 (range − 2.4–1.3); *LEP-/-* (*n* = 25): mean 5.5 (range 2.7 − 12.5); **B** Adults: *LEP wt/wt* (*n* = 4): mean 0.3 (range − 1.0–1.0); *LEP wt/-* (*n* = 64): mean 1.0 (range − 0.9–4.2); *LEP-/-* (*n* = 13): mean 4.8 (range 4.1–5.2); **C** BMI *z* scores in association with *LEP* variant (green circle: *wt/wt* subject; blue square: *wt/-* subject; red triangle: *-/-* subject; yellow line: BMI *z* score = 1) (subjects reported in different studies, but showing the same variant are grouped together. It is not distinguished between children and adults. Only variants reported to be carried by *wt/-* are shown. However, not always a BMI *z* score for the *wt/-* carrier could be calculated; *p < 0.05)

*BMI z-scores in LEP heterozygotes vs WT and LEP homozygous relatives:* We plotted the calculated BMI *z* scores for the *LEP wt/-*, *wt/wt*, and *LEP -/-* relatives depending on the variant position (Fig. 2A–C). The individual BMI *z* scores for *LEP wt/-* are comparable to the BMI *z* scores of the *wt/wt* relatives. The highest BMI *z* score values for *LEP wt/-* were reported in adults showing variants p.V110M and p.H118L in the studies of Karvonen et al. and of Zhao et al., who did not report *wt/wt* and *-/-* relatives [38, 89]. We did not find differences between BMI *z* scores in male and female adults with *LEP wt/-* (data not shown). Only male children are reported in literature; thus, comparison with female children was not possible.

*Body fat percentage in LEP heterozygotes vs. WT:* In two articles reporting on variant p.G133Vfs*15, information on body fat (BF) percentage in *LEP wt/-*as well as in *wt/wt* controls was available [19, 32], whereby double reporting cannot be excluded. The calculated BF percentage was higher in *LEP wt/-* (5 including four obese adults and one child) compared to *wt/wt* relatives (3 children) (mean 33% (range 15–43%) vs. mean 17% (range 14–20%) [19, 32]. In a study of Farooqi et al., BF percentage measured by X-ray absorptiometry was reported to be significantly higher than predicted by the Deurenberg formula in 13 subjects carrying *wt/- LEP* variants (measured: 41.4% vs. predicted: 34.4%), while in six *wt/wt* subjects, the calculated BF was found to be similar to predicted values [32].

*Body fat percentage in LEP heterozygotes vs. control group:* Compared to BF in a control group of 5 individuals without known genotype, BF in two *LEP wt/-* (mean 17.6 vs 16.5%) was similar [80].

**Fig. 3:**
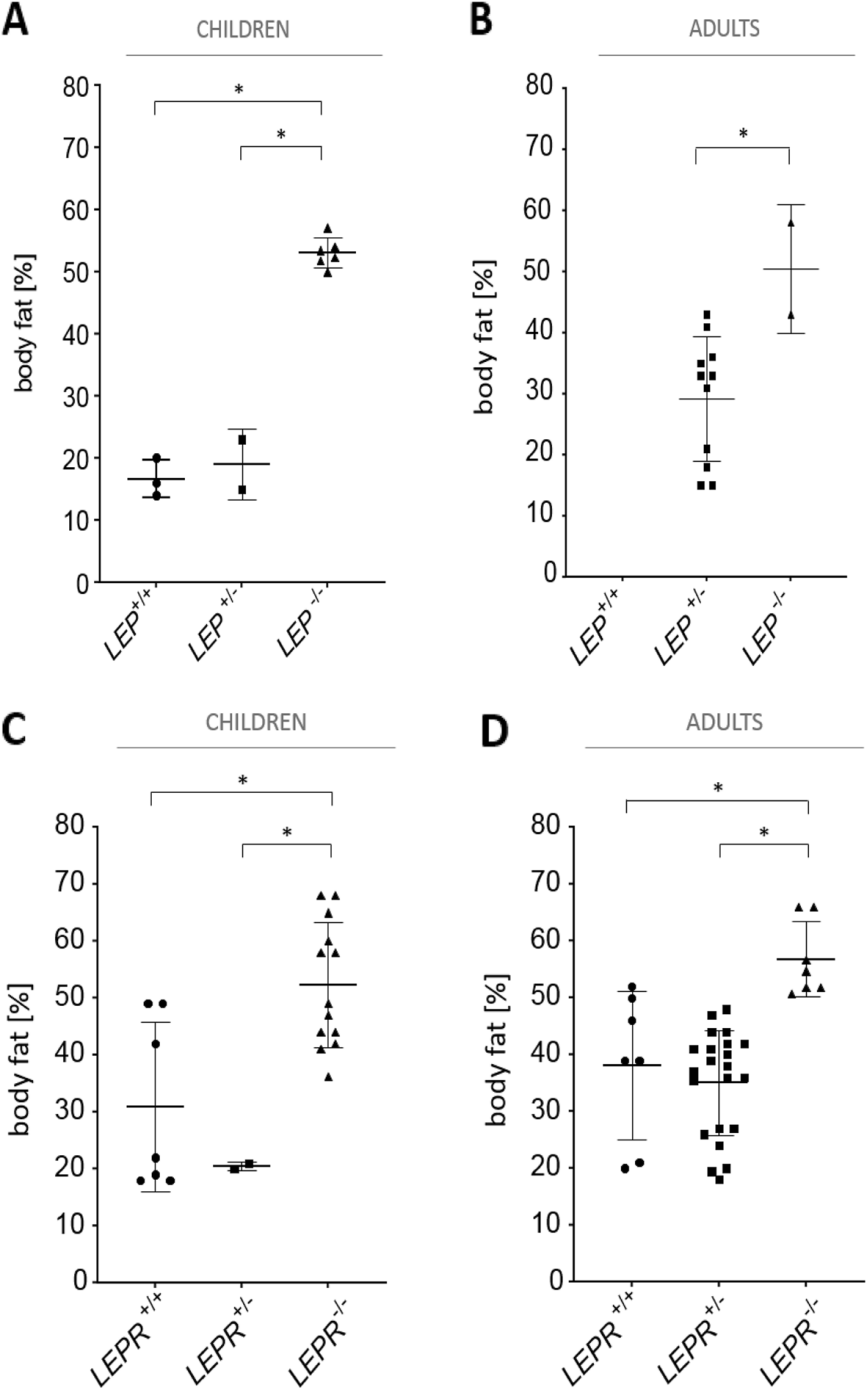
Illustration of individual body fat (BF) values [%] for children and adults in association with genotype for *LEP* (**A**, **B**) or *LEPR* (**C**, **D**) (*wt/wt* vs. *wt/-* vs. *-/-*; *p < 0.05). **A** Children: *LEP wt/wt* (*n* = 3): mean 17% (range 14–20%); *LEP wt/-* (*n* = 2): mean 19% (range 15–23%); *LEP*-/- (*n* = 6): mean 53% (range 50–57%). **B** Adults: *LEP wt/wt* (*n* = 0) *LEP wt/-* (*n* = 11): mean 28% (range 15–36%); *LEP*-/- (*n* = 2): mean 50.4% (range 42.8–57.9%); **C Children: ***LEPR wt/wt* (*n* = 7) mean 31% (range 18–49%); *LEPR* wt/- (*n* = 2): mean 20.5% (range 20–21%); *LEPR*-/- (*n* = 13): mean 54% (range 41–68%); **D** Adults: *LEPR wt/wt* (*n* = 7) mean 38% (range 20–52%); *LEPR wt/-* (*n* = 22) mean: 35% (range 18–48%); *LEPR*-/- (*n* = 7): mean 57% (range 50.7–66%)

*Body fat percentage in LEP heterozygotes vs WT and LEP homozygous relatives:* We plotted available individual BF values for *LEP wt/-*, *wt/wt*, and *LEP -/-* subjects in children and adults (Fig. 3A, B). Comparison between *LEP wt/-*and *wt/wt* adult subjects was not applicable due to limited data, but in children, BF percentage was similar in *LEP wt/-* and *wt/wt* group.

*Leptin serum concentrations in LEP heterozygotes vs. WT or control group:* Leptin levels in *LEP wt/-* and *wt/wt* relatives were provided in six studies [19, 22, 32, 37, 80, 82]. A further study reported leptin values also in a not-related *wt/wt* control group [88]. In five out of these seven studies, leptin levels were similar in *wt/-* and *wt/wt* subjects (Table S2). In the study of Farooqi et al. [32], leptin levels were lower in *LEP wt/-* than in *wt/wt* subjects (mean 5.5 ng/ml vs mean 27.7 ng/ml), whereas they were higher in the study of Saeed et al. (mean 6.9 ng/ml vs mean 3.5 ng/ml) [88]. In both studies, the reported *LEP* variant was p.G133Vfs*15, which leads to a secretion defect. Compared to leptin levels in the control groups without known genotype, leptin levels in *LEP wt/-* were higher in one study [86] and slightly lower in another study [87]. In these two studies, variants p.I35del and p.R105W were reported.

**Fig. 4:**
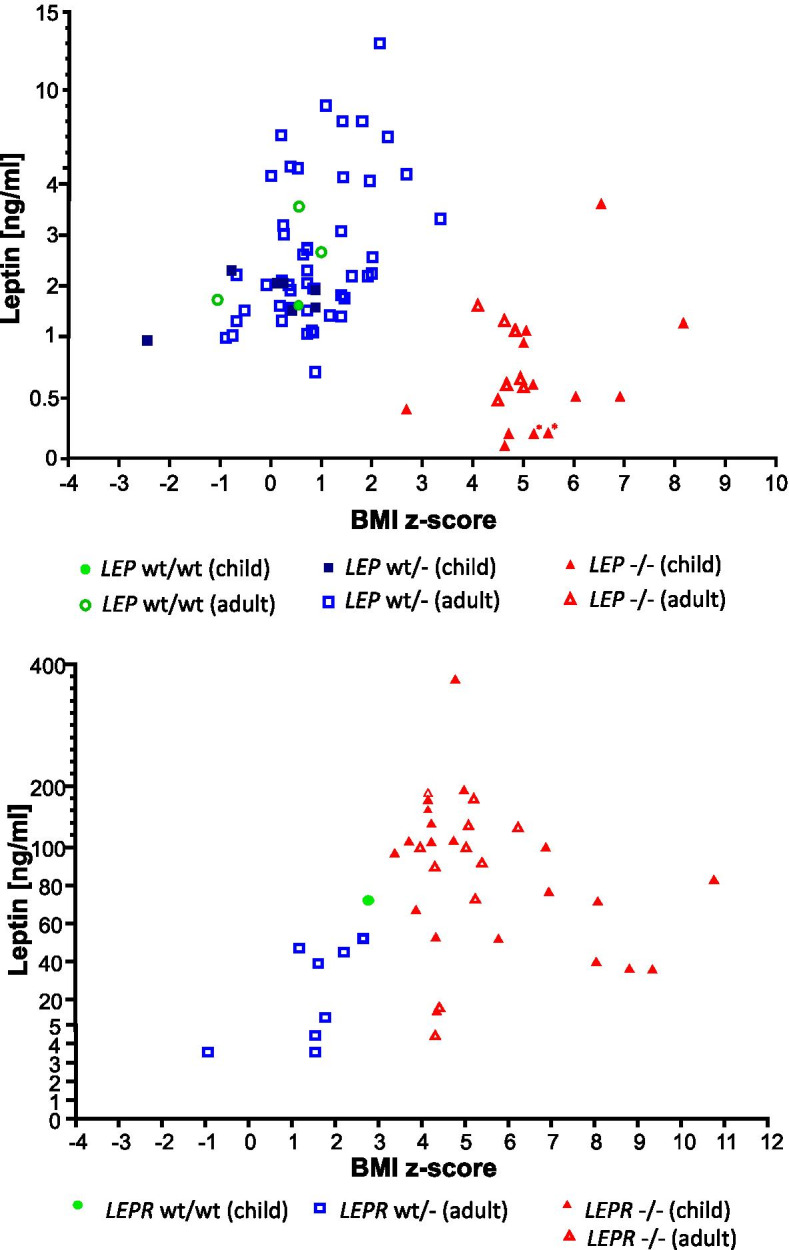
Leptin levels [ng/ml] in relation to BMI *z* score in *LEP* (**A**) and *LEPR* (**B**) variant carriers and wildtype subjects (*wt/wt* vs. *wt/-* vs. *-/-*). Data are shown for children and adults

*Leptin levels in LEP heterozygotes vs WT and LEP homozygous relatives*: We calculated a mean leptin value of 4.6 ng/ml (range 0.7–24 ng/ml) in 70 *LEP wt/-* adults. In 16 WT adults mean leptin level was 7.6 ng/ml (1–32 ng/ml). In 6 *LEP wt/-* children mean leptin was 2.1 ng/ml (range 1.5–3 ng/ml), while in 6 *wt/wt* obese control children 32.8 ng/ml (1.6–133.1 ng/ml) [90]. Differences in leptin levels both in the adult and in the children *LEP wt/-* vs *wt/wt* group were not significant (*p* > 0.05). In the homozygous *LEP* group, mean leptin was significantly lower (*p* < 0.05) than in *LEP wt/-* and *wt/wt* adults, with a mean of 0.9 ng/ml in 12 adults (range 0.5–1.6 ng/ml). Mean leptin in *LEP -/-* children (0.9 ng/ml in 19 children (range: not detectable leptin -3.6 ng/ml)) was significantly lower than in *LEP wt/-* children (*p* < 0.05). We could not analyze leptin levels in relation to body fat percentage, but only in relation to BMI *z* score (Fig. 4A). In *LEP wt/-*, higher leptin levels than in *LEP -/-* are detected, although they show lower BMI *z* score than homozygous subjects. In two studies describing *LEP wt/-*with severe obesity not reporting *wt/wt* subjects, very low leptin levels in relation to high BMI (4.6 ng/ml, 3.3 ng/ml) [38] or non-detectable leptin [89] were observed.

*Metabolic parameters in LEP heterozygotes vs. WT:* Three studies reported the metabolic phenotype in *LEP wt/-* as well as in *wt/wt* related subjects. We found a higher frequency of metabolic abnormalities in *LEP wt/-* than in *wt/wt* subjects, including hypercholesterinemia, hyperinsulinemia, and hypertriglyceridemia in two studies [22, 82]. However, since these abnormalities were not always present in *LEP -/-* subjects, this finding may not be related to a pathogenic effect of *LEP* variants. One study comparing *LEP wt/-* to non-related *wt/wt* subjects did show abnormalities neither in *LEP wt/-* nor in *wt/wt* [88]. One further study indicating the relationship of leptin secretion and TSH circadian secretion pattern observed a weaker correlation between leptin and TSH secretion pattern in *LEP wt/-* than in *wt/wt* subjects [80]. Furthermore, metabolic changes were observed in one severely obese subject with the *LEP wt/-* variant p.H118L, who showed hypertension, metabolic syndrome, and hepatic steatosis. Here, no WT relatives were described [89]. Thus, in several studies among the few ones providing metabolic parameters, metabolic abnormalities were found in *LEP wt/-* subjects, but causal association to leptin variant status is unclear, since metabolic abnormalities were not always present in *LEP -/-* subjects.

*Metabolic parameters in LEP heterozygotes vs. control group:* In two studies, *LEP wt/-* were compared to the control groups without known genotype. In the first one, we observed no metabolic abnormalities in *LEP* heterozygotes in comparison to controls [87]. In the second one, hyperinsulinemia was found in one out of three *LEP wt/-*, but not in the control group [86].

*Eating behavior in LEP heterozygotes:* In the reviewed studies, no standardized assessment of hyperphagia in *LEP* heterozygotes has been performed. Two studies reported the absence of hyperphagia in *LEP wt/-* [35, 86]. In the case study of Murray et al. reporting the p.P23R variant with supposedly increased activity, two heterozygous subjects with either underweight or low weight showed low appetite, while one heterozygous subject showed normal appetite [81].

*Immunological phenotype in LEP heterozygotes:* We only found two studies commenting on immunological aspects of *LEP wt/-*. One study investigated the activity of antioxidant enzymes and plasma levels of selenium, zinc, and manganese and found them decreased both in *wt/-* and *-/- LEP* subjects in comparison to control subjects [87], wherefore the authors suggested a role of leptin signaling in regulation of antioxidant defense system. Furthermore, a prolonged recovery after viral illness was observed in the case of the subject showing the p.P23R variant with underweight and delayed puberty [81].

*Puberty and reproductive function in LEP heterozygotes:* In most studies, no information about pubertal development in *LEP* heterozygous subjects was provided. In one study, normal pubertal development [82] was reported in *LEP* heterozygotes. In a subject showing the *LEP wt/-* variant p.P23R and a reduced body mass index, delayed puberty was observed. His mother and half-brother, carrying the same variant, showed delayed menarche and normal pubertal development, respectively [81]. In a further study, normal reproductive function was reported in *LEP wt/-*, while *LEP -/-* variant carrier in the same family never entered puberty [85]. We assume that fertility was not severely impaired in *LEP wt/-*parents with *-/-* children.

The following indications for phenotypic effects of *LEP* heterozygosity in humans were found:

- Qualitative and quantitative reporting of weight status in reviewed studies suggests higher frequency of overweight and obesity in adult *LEP wt/-* vs *wt/wt*
- Two out of three studies comparing BF percentage showed a higher BF percentage in *LEP wt/-* than in *wt/wt*
- No strong evidence for lower leptin levels in *LEP wt/-* compared to *wt/wt* was found
- A higher frequency of metabolic changes including hyperinsulinemia and dyslipidemia was observed in *LEP wt/-* than in *wt/wt*, but causality of *LEP* variant status for these conditions remains unclear
- *LEP* heterozygosity does not seem to strongly impair eating behavior, pubertal development, or reproductive and immunological function, since these conditions are not considered in most reports

### Phenotype in human carriers of *LEPR wt/*- variants

We found 14 studies reporting in total 108 carriers of *LEPR* heterozygous variants 90 adults, 11 children, and 7 subjects with no reported age, describing in total 35 different *LEPR* variants. Among the 108 carriers, 41 were male subjects and 53 female subjects, whereas in further 14 individuals, sex category was not reported. Out of 90 adults exhibiting *LEPR* heterozygous variants, 26 were reported as normal weight, 26 as overweight, and 21 as obese, and in 17, information was missing. Among eleven *LEPR wt/-* children, three showed normal weight and six obesity (double reporting cannot be excluded in the cases with obesity). In two *LEPR wt/-* children, weight status was not reported. In further seven *LEPR wt/-* subjects with no reported age normal weight was reported in one individual, whereas in the other six individuals, information was missing.

*Weight status in LEPR heterozygotes vs WT relatives:* In seven studies, weight status in *LEPR wt/-* subjects as well as in *wt/wt* relatives was provided, at least for some of the reported families [20, 36, 37, 91–94] (Table S3). In these seven studies, no overall increased frequency of overweight or obesity in in *LEPR wt/-* vs *wt/wt* relatives was found. In the heterozygous carriers of two specific variants, p.R612H and p.M585Dfs*2, obesity was reported, whereas normal weight or overweight was found in *wt/wt* relatives [91, 92].

**Fig. 5:**
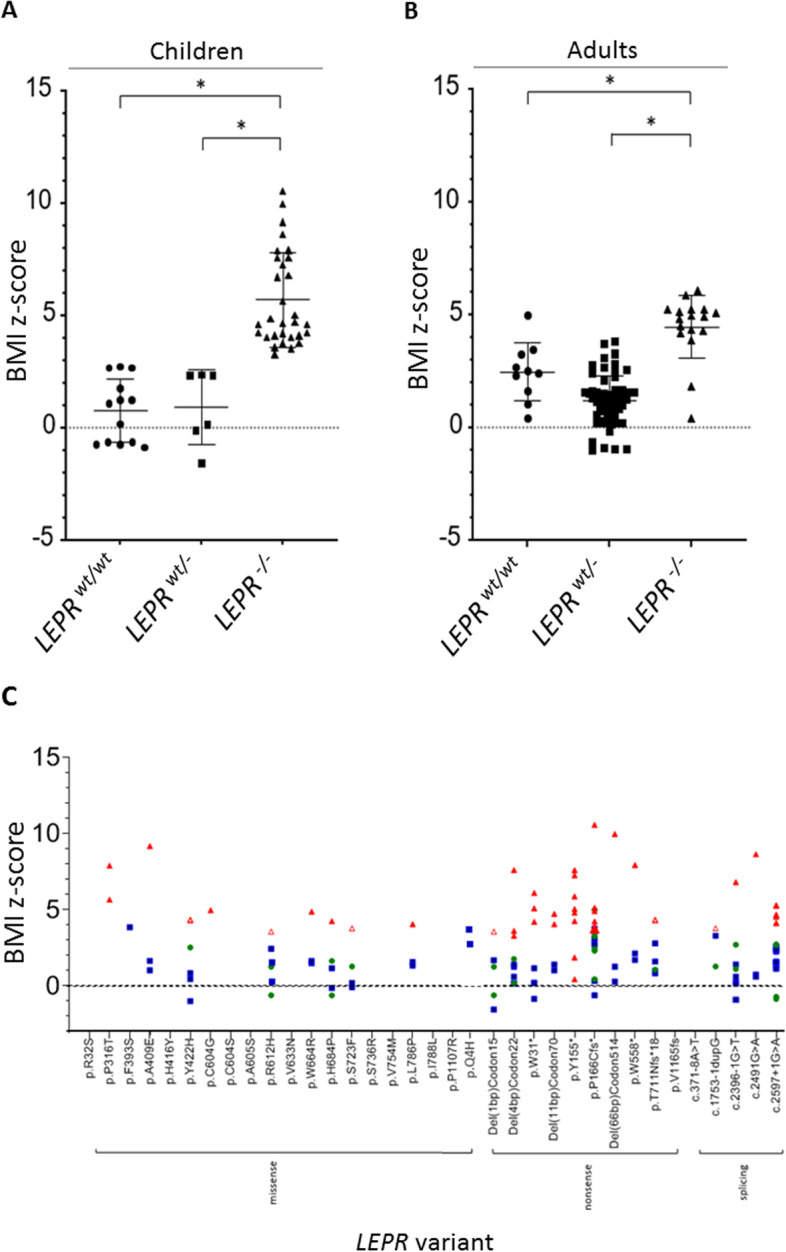
**A**, **B** Illustration of individual BMI *z* scores in children (**A**) and adults (**B**) depending on *LEPR* genotype (*wt/wt* vs. *wt/-* vs. *wt/-*; *p ≤ 0.05). **A** Children: *LEPR wt/wt* (*n* = 13): mean 0.5 (range 0.4–0.6); LEPR *wt/-* (*n* = 7): mean 0.9 (range − 1.6–2.4); *LEPR*-/- (*n* = 17): mean 5.7 (range 3.3–10.6); **B** Adults: *LEPR wt/wt* (*n* = 10): mean 2.5 (range 0.4–5.0); *LEPR* wt/- (*n* = 60): mean 1.2 (range − 1.0–3.8); *LEPR*-/- (*n* = 30): mean 4.5 (range 0.4–6.1); **C** BMI *z* scores in association with *LEPR* variant (green circle: *wt/wt* subject; blue square: *wt/-* subject; red triangle: *-/-* subject; unfilled red triangle: compound *wt/-* subject) (Only variants reported to be carried by *wt/-* are shown). However, not always a BMI *z* score for the *wt/-* carrier could be calculated. It is not distinguished between children and adults. Subjects reported in different studies, but showing the same variant are grouped together. If *wt/wt* subjects in one family with two different *wt/-* variants were described, they BMI *z* scores were plotted for both variants)

*BMI z score in LEPR heterozygous vs WT or LEPR homozygous subjects:* We plotted the calculated BMI *z* score values to show the distribution within *LEPR wt/-*, *wt/wt*, and -/- groups in association with variant position (Fig. 5A–C). Here, no significant difference in BMI *z* score was observed between *LEPR wt/-* and *wt/wt* subjects. It should be considered that 8 out of the 10 *wt/wt* adults were described within French Reunion Island families, which were reported to be “prone to obesity” [93]. We did not find significant differences between BMI *z* scores of male and female adults (data not shown). Comparison between male and female children was not possible, because only one female child was reported.

*Body fat percentage in LEPR heterozygotes vs. WT:* Four studies compared body fat (BF) percentage in *LEPR wt/-* and *wt/wt* subjects [20, 36, 91, 93]. BF percentage was measured via biphotonic absorptiometry [20, 37] or X-ray absorptiometry [91, 93]. In three of these four studies, no differences in BF between *LEPR wt/-* and *wt/wt* relatives were found. In one study by Farooqi et al., the absolute difference in measured vs. predicted percentage of BF was reported to be significantly higher in *LEPR wt/-* than in *wt/wt* relatives (8.2% vs 2.1%) [91].

*Body fat percentage in LEPR heterozygotes vs. WT vs homozygous subjects*: We plotted BF values in *LEPR wt/-* versus *wt/wt* and *-/-* group (Fig. 3C, D). BF in *LEPR wt/-* did not differ significantly from BF in adult *wt/wt* subjects. In children, the sample size was too small to prove significance.

*Leptin serum concentrations in LEPR heterozygotes vs. WT:* Information on leptin values in *LEPR wt/-* as well as in *wt/wt* controls was provided in three studies describing the *LEPR* variant G > A in exon 16 [20, 36, 37]. The first study [20] described very high leptin levels in carriers of the *LEPR* variant, which was later shown to lead to a truncated LEPR protein binding to leptin in the blood and thus rising measured leptin values. The following two studies [36, 37] reporting the same variant additionally measured free leptin values. We considered therefore only the free leptin values for our calculations [36, 37]. In these two studies, free leptin in *LEP wt/-* were not higher than in related *wt/wt* children.

*Leptin serum concentration in LEPR heterozygotes vs WT vs homozygous subjects:* We compared leptin values between the *LEPR wt/-*, *wt/wt*, and *-/-* group. Adult *LEPR wt/-* carriers had mean leptin levels of 28.8 ng/ml (range 2.2–52 ng/ml, 12 subjects). No leptin values were reported for *wt/wt* adults and for *LEPR wt/-* children in reviewed studies. In one obese *wt/wt* child, circulating leptin was 72.2 ng/ml [36], while in a group of normal weight *wt/wt* children (*n* = 11), mean leptin values of < 10 ng/ml had been reported [95]. In subjects homozygous for *LEPR* variants, leptin levels were higher than in heterozygous subjects. Eleven *LEPR -/-* adults showed a mean leptin level of 92.4 ng/ml (range 4.4–180 ng/ml) and 21 children showed a mean leptin level of 105.1 ng/ml (range 14–365 ng/ml). Since body fat was only provided in a few cases, we plotted leptin levels of the *LEPR wt/-*, *-/-*, and *wt/wt* group in association to BMI *z* score (Fig. 4B). Comparison to *wt/wt* within the same age group was not possible due to limited data.

*Metabolic abnormalities in LEPR heterozygotes vs WT or LEPR homozygous subjects:* Information on metabolic parameters or on comorbidities in heterozygotes was provided in four studies [20, 93, 94, 96]. In two of them [20, 93], *LEPR wt/-* with a G- > A exchange in donor site of exon 16 or deletion of exon 6–8 could be compared to *wt/wt* relatives. Here, we found a slightly higher rate of dyslipidemia in *LEPR wt/-* than in *wt/wt* subjects (Table S3). These findings were not always present in related *LEPR -/-*. In two further studies metabolic parameters were reported in *LEPR wt/-* without being compared to parameters in related *wt/wt* subjects. In the case of the patient with severe obesity showing heterozygous *LEPR* variant p.F393S [96] diabetes type 2, hypertension and metabolic syndrome were reported. In the carrier of the p.799-1G > T *LEPR* heterozygous variant, no metabolic abnormalities were reported [94]. Thus, in most studies providing information on metabolic parameters, abnormalities were found in *LEP wt/-*, but causality of *LEPR wt/-* is questionable.

*Eating behavior in LEPR heterozygotes:* Hyperphagia in *LEPR wt/-* was not described in the evaluated studies.

*Immunological phenotype in LEPR heterozygotes:* One study reported no increased susceptibility for infections [97]. The other 12 reviewed studies reported no information about immunological status of *LEPR wt/-*.

*Puberty and reproductive function in LEPR heterozygotes:* Two studies provided information on pubertal development in *LEPR wt/-*. In one study, normal sexual maturation or normal reproductive function in four *LEPR wt/-* were reported. In one case, a female subject had menarche with 15 years [20]. In the study on families from French Reunion Island, information about one family with the p.(P166Cfs*) variant was provided [93]. No hypogonadism was observed in five *LEPR wt/-* from this family, while in three out of five related subjects who were -/- for this variant, hypogonadism was observed. As for *LEP wt/-* parents, not severely impaired reproductive function can be assumed for *LEPR wt/-* parents.

The following indications on effects of *LEPR* heterozygosity in humans were found:

- There were some indications for higher frequency of overweight or obesity in *LEPR wt/-* compared to *wt/wt* relatives
- One study reported significantly higher than predicted BF measured by X-ray absorptiometry in *LEPR* heterozygotes [91]. In three articles reporting BF measured by biphotonic absorptiometry or X-ray absorptiometry, this finding was not confirmed [20, 36, 93]
- Leptin levels in *LEPR wt/-* could not be compared to leptin levels in *wt/wt* in the same age group, and information on associated BF was scarce
- Based on two studies [20, 93], dyslipidemia seemed to be more frequent in *LEPR wt/-* than in *wt/wt* subjects
- No report on hyperphagia, immunological disease, or impaired pubertal development was found in *LEPR* heterozygotes

## Discussion

This systematic review summed up the phenotype in *LEP* and *LEPR* heterozygosity, considering animal and human studies. We aimed to identify a specific phenotype in regard to weight status, body fat percentage, leptin levels, and metabolic parameters by comparing reported data in carriers of highly penetrant *LEP/LEPR* variants vs *wt/wt*. In our review, we show that the published literature provides evidence for intermediate phenotypic effects in *LEP/LEPR* heterozygosity both in animal and human studies: (1) Evidence for different weight status in *LEP/LEPR* heterozygosity vs *wt/wt* was found in both animals and humans. (2) Animal studies and a few human studies suggest higher body fat percentage in *LEP* and *LEPR* heterozygotes compared to *wt/wt*. (3) Lower leptin levels in *LEP* heterozygous animals, and higher leptin levels in *LEPR* heterozygous animals vs *wt/wt* were found. In humans, there was no consistent evidence for different levels in heterozygotes vs *wt/wt*, but only limited data was available. (4) We noticed metabolic abnormalities in a fraction of reports on animal and human *LEP* and *LEPR* heterozygotes compared to *wt/wt*, but causal association with *LEP* or *LEPR* heterozygosity remains unclear.

### Weight status in *LEP* and *LEPR* heterozygosity vs WT

We found hints supporting the assumption of increased weight status in heterozygosity for *LEP/LEPR* vs *wt/wt* both in animal and human studies. In animal models, only a part of the studies investigating weight status found differences between heterozygous and *wt/wt* animals, whereas others did not observe significant differences. Conflicting results may be depending on different genetic backgrounds of animal models, age, sex, and different experimental settings used, including pathogen-free rearing. Moreover, it needs to be further investigated, if parental origin of *LEP* or *LEPR* heterozygosity may lead to different phenotype in offspring. In offspring of *LEPR wt/-* animals, higher weight than in offspring of *wt/wt* was observed in two studies [59, 63], whereas in one study, offspring of *LEPR wt/-* showed decreased weight if compared to offspring of *wt/wt* [61]. 28% of the adult human subjects carrying *LEP* heterozygous variants were overweight and 16% were obese. 43% of the adult human subjects carrying *LEPR* heterozygous variants were overweight and 20% were obese. Heterozygous variants were often reported without comparison to *wt/wt* subjects, so that causality of the association of *LEP/LEPR* heterozygous variants with higher weight status often remained uncertain. We compared the phenotype in *LEP* and *LEPR wt/-* vs *wt/wt* relatives within described families but did not find conclusive evidence for significant differences (Table S2, 3, Figs. 2C and 5C). Higher BMI *z* scores than in *wt/wt* relatives were found in carriers of *LEP* variants p.G133Vfs*15 and p.C117F and of *LEPR* variants p.R612H and p.M585Dfs*2. The *LEP* p.P23R variant with a putatively increased leptin activity was reported in two underweight heterozygous subjects [81]. This may indicate an influence of *LEP/LEPR* heterozygosity on weight status, similarly to reported gain-of function *MC4R* variants which are associated with lower BMI in humans [98, 99].

Importantly, the majority of individuals who showed *LEP* or *LEPR wt/-* variants and increased weight status were adults. Onset of overweight or of obesity in *LEP* and *LEPR wt/-* was reported to occur during adulthood in previous studies [92, 93, 96, 100]. This observation may be unspecific, since prevalence of obesity in general population is higher in adults than in children. Moreover, a bias in identification of heterozygotes should be taken into account, because mainly parents of *LEP* or *LEPR -/-* children and thus preferentially adult individuals with *LEP* or *LEPR wt/-* have been reported. Nevertheless, as proposed in animal studies, diet, sex, and other factors [101, 102], such as age may influence development of obesity in *LEP* and *LEPR* heterozygotes. It is plausible that in an obesogenic environment, individuals heterozygous for *LEP* or *LEPR* variants may preferentially develop obesity with increasing age if compared to *wt/wt* subjects.

### Evidence suggesting higher body fat percentage in *LEP* and *LEPR* heterozygotes compared to WT

We found evidence suggesting higher body fat (BF) percentage in *LEP* and *LEPR* heterozygous animal models vs *wt/wt*. In humans with heterozygous *LEP* or *LEPR* variants, there was only limited data on BF percentage. Extensive studies by Farooqi et al. investigating differences in human heterozygous subjects for *LEP* and *LEPR* had shown higher than predicted BF in heterozygotes than in *wt/wt* subjects [32, 91].

It was proposed that low leptin levels lead to an increase in body fat, in an attempt of the body to restore a certain set point in stored energy [32]. In that sense, low leptin values provide a signal of energy deficit to the CNS, representing a “starvation signal” [103, 104]”. Body fat reduction after therapeutic leptin substitution in individuals heterozygous for *LEP* may be expected [105].

### Difference in leptin levels in *LEP* heterozygotes versus WT and possible implications

Animal studies showed evidence for lower leptin levels in *LEP* wt/- compared to *wt/wt* animals, especially in relation to fat mass, whereas in humans, we did not find conclusive data. It is plausible that both *LEP* alleles are expressed equally in heterozygotes. Thus, 50% reduced levels of leptin could be expected in *LEP* wt/-compared to *wt/wt* subjects with corresponding body mass, fat mass, and age would be expected. This assumption is supported by the finding of bioactive and inactive leptin in a ratio of 0.5 in carriers of heterozygous variant p.D100Y and p.N103K [35]. However, we did not find strong evidence confirming the assumption of reduced leptin in *LEP wt/-* vs *wt/wt* in other studies. We want to point out though that very limited data were accessible on *wt/wt* relatives and missing data on associated body and fat mass must be considered. It has been speculated that low leptin in *LEP* heterozygotes may lead to increased fat mass, as an attempt of the body to increase leptin production, in a sort of feedback regulation mechanism [32]. In Pima Indians, low leptin precedes weight gain [106]. It seems plausible that the homeostatic system shows higher sensitivity for falling leptin levels than for high leptin levels, to prevent the condition of starvation. Partial leptin deficiency may thus lead to increased body fat storage and high BMI. If leptin levels in *LEP wt/-* are confirmed to be low in relation to their body fat, *LEP* heterozygosity may represent a condition of relative leptin deficiency, implying possible therapeutic options in *LEP* heterozygotes showing overweight or obesity.

### Difference in leptin levels in *LEPR* heterozygotes versus WT and possible implications

We found evidence for higher leptin levels in *LEPR* wt/- animals. Here, binding of leptin to circulating truncated leptin receptor cannot be excluded, since free leptin values were not provided. However, hyperleptinemia was directly proportional to leptin mRNA in fat tissue in one study [78]. Assessment of leptin levels in humans carrying *LEPR* variants was limited, due to small sample size in *wt/wt* and no possibility of comparison within the same age groups. Since binding of leptin to truncated receptor was excluded in the considered human variants, high leptin levels may be due to a compensatory overproduction of leptin to overcome receptor impairment. The homeostatic system seems to respond more sensitively to falling leptin than to increased leptin levels. Increased leptin levels in *LEPR* heterozygosity thus seem to be the consequence of defective feedback regulation and have the aim of achieving adequate post-receptor signaling, as proposed by Chung et al. [41]. However, high leptin may also be due to higher body fat percentage, which was described in the study of Farooqi et al. [91]. Melanocortin-4 receptor agonism has been shown to reduce weight in leptin receptor deficiency in individuals carrying *LEPR -/-* variants [107]. If, as we suspect, leptin receptor signaling is impaired in *LEPR* heterozygosity, treatment of pathways downstream of leptin receptor may represent possible therapeutic approaches in the future.

### Association of *LEP* and *LEPR* heterozygosity with metabolic abnormalities remains unclear

If present, both partial leptin deficiency in *LEP* heterozygotes and impaired receptor signaling in *LEPR* heterozygotes should result in insufficient activation of pathway downstream of leptin. Since leptin insufficiency is associated with metabolic disease and insulin resistance, we would expect metabolic abnormalities in *LEP/LEPR wt/-*. However, we found conflicting results on metabolic abnormalities in *wt/-* vs *wt/wt* animals. Differences may be due to different experimental settings as well as to varying sex and age of the animal model used. Importantly, several studies on heterozygous *LEP* mice showed increased metabolic impairment than in *wt/wt* under high-fat diets, so that increased disposition to metabolic disease can be assumed under adipogenic conditions [40, 49, 51]. One study observed that *LEPR wt/-* mice, unlike *LEPR -/-* mice, did not develop diabetes due to a compensatory mechanism leading to suppression of beta-cell apoptosis [57]. This finding suggests that compensatory mechanisms supporting healthy metabolism may be activated in *LEP/LEPR* heterozygosity. On the other side, fatty liver was observed in *LEPR wt/-* rats even in the absence of obesity. The authors proposed that peripheral disruption of leptin action, even in case of heterozygosity may lead to triglyceride accumulation in the liver [69]. Moreover, a gene-dosage effect in lipogenic activity in adipocytes [68] and in pancreatic release of insulin during glucose perfusion [66] was reported in animal studies, which would support intermediate effects of *LEP/LEPR* heterozygosity on metabolism.

In humans heterozygous for *LEP* and *LEPR*, we observed a slightly increased frequency of metabolic abnormalities in comparison to WT relatives. Association of the metabolic abnormalities with heterozygous *LEP* or *LEPR* status remains unclear, since abnormalities were not always found in related homozygotes. It is conceivable that metabolic abnormalities in *LEP/LEPR* heterozygosity may be secondary to overweight or obesity, irrespective of the genetic status, is conceivable. To clarify causality of metabolic abnormalities, metabolic features in *LEP* and *LEPR* heterozygotes prior to and after loss of body fat should be investigated, as already proposed [108].

### No evidence for effects of *LEP* and *LEPR* heterozygosity on eating behavior, immunological phenotype, and puberty

Hyperphagia was reported in pregnant *Lepr wt/-* mice [65], but several other studies did not confirm abnormal eating behavior in these animals [69, 72]. Only few reports on human subjects commented on eating behavior of *LEP* heterozygotes, reporting either no hyperphagia [35, 81, 86] or reduced appetite, as observed in two subjects with the *LEP* variant p.P23R leading to putatively increased activity [81]. In one study on binge-eating in severe obesity associated with *MC4R* and *LEPR* variants, it was observed that prevalence of binge-eating in *LEPR* heterozygous variant carriers, including p.R612H variant, was higher than in controls, but no individual cases were reported [109]. We did not find evidence for an impairment of the immune system in *LEP* or *LEPR wt/-*. Furthermore, reviewed animal and human studies did not provide evidence for impaired puberty or reproductive dysfunction in *LEP/LEPR* heterozygosity. Even if *Lepr wt/-* mice were used as a model for gestational diabetes over many years [61, 65], a recent study could not confirm gestational diabetes phenotype in *Lepr wt/-* mice [60]. The fact that a major fraction of reported *LEP* and *LEPR* heterozygotes were parents of *-/-* children speaks against severe reproductive dysfunction in heterozygotes.

### Comments on effects of mono-allelic likely pathogenic variants in *MC4R* and in proteins structurally similar to *LEP*-*LEPR*

In heterozygosity, alleles may behave as codominant, dominant negative, or haploinsufficient with regard to resulting phenotype. In codominance, both alleles are expressed with the consequence of an intermediate phenotype. Dominant negative effects consist in disruption of *wt/wt* protein or competition with *wt/wt* protein, while haploinsufficiency is found in mutations leading to reduced gene expression or reduced protein activity [110]. In a recent article on the effects of *wt/-* vs *-/- MC4R* variant p.E42*, severe early onset obesity was observed in homozygous individuals, but heterozygotes showed overweight or no difference in phenotype if compared to *wt/wt* family members [111]. The authors suggested that haploinsufficiency may be the underlying cause, meaning that *MC4R* needs both alleles to fully express the *wt/wt* phenotype [111]. Growth hormone (GH) and its receptor (GHR) show structural similarities to LEP-LEPR, and both GHR and LEPR both form homodimers after ligand binding [112]. In a recent study on a large pediatric cohort with GH deficiency and short stature, the authors observed that a biallelic deletion was associated with the most severe end of phenotypic spectrum. However, the most common mutations found were heterozygous GH mutations resulting in GH deficiency [29]. Heterozygous *GHR* variants seem to cause growth impairment, and in the study of Porto et al., authors proposed that *wt/-* variants may lead to only 25% of receptors interacting adequately with GH [30]. If both *LEPR* alleles are expressed equally, it is well conceivable that similar to GHR *wt/-* variants, *wt/-* variants in *LEPR* associated with structural changes may affect 75% of receptors. Also G-CSF and its receptor belong to the same family as LEP-LEPR [112]. In the case of *wt/-* variants in *G-CSF*, dominant-negative effects affecting *wt/wt* receptors have been proposed [113]. Colocalization of mutated and *wt/wt* receptor has been demonstrated [114]. Another study postulated that CSF-1 receptor mutations affect phenotype by haploinsufficiency [31]. Haploinsufficiency has also been postulated in heterozygous mutations in partial prohormone convertase 1 (*PCSK1*) leading to obesity [115]. Also, dominant-negative effects have been proposed in PCSK mutants which may influence WT PCSK by formation of heteroduplexes which are targeted for proteosomal degradation [116]. Codominant effects in earliest days of life were proposed in *LEPR wt/-* rats showing an intermediate phenotype between *wt/wt* and *fa* homozygous rats with regard to body fat and body weight. The authors suggested that in heterozygotes, functional, and mutant LEPR may act in a competitive manner or that LEPR production may be too low to ensure adequate LEPR functionality [102]. Later, Schwarzer et al. stated that the effect of *LEPR* genotype on body fat changed from being codominant to partially recessive with age of the rats. Fat mass changed with age and differed between *wt/-* and *wt/wt* to a lesser extent than between *wt/-* and *-/-* [75].

Thus, similar to structurally related proteins, also *wt/-* variants in *LEP* and *LEPR* may lead to phenotypic changes. Hence, haploinsufficiency can provide a plausible explanation for the slight phenotypic effects observed in *LEP* and *LEPR* heterozygotes. If the assumption of phenotypic effects in *LEP* and *LEPR* heterozygosity will be confirmed, recessive inheritance of *LEP* and *LEPR* associated disease should be questioned.

### Phenotype in *LEP wt/*- variants versus phenotype in *LEPR wt/*- variants

In this analysis, we did not find strong evidence for differences in *LEP* vs *LEPR wt/-* phenotype. Differences may be expected (1) if leptin signaling is achieved other than through activating the leptin receptor, thus leading to a more impaired phenotype in (partial) leptin deficiency than in (partial) leptin receptor deficiency. On the other hand, differences may also be expected (2) if leptin receptor function independent of the classic metabolic signaling as described in [117] is considered, thus putatively leading to more severe impairment in (partial) leptin receptor deficiency than in (partial) leptin deficiency, regardless of leptin values. However, if the highly interwoven function of leptin and leptin receptor are considered, no consistent differences should be expected in the phenotype of *LEP* vs *LEPR wt/-* phenotype. To our knowledge, no studies have been performed investigating differences of phenotype in *LEP* vs *LEPR -/-* subjects. This topic should be a future subject of investigation.

### Mono-allelic likely pathogenic variants of *LEP* and *LEPR* may provide evolutionary advantages

Partial leptin and leptin receptor expression and activation of central and peripheral compensatory mechanisms in *LEP*/*LEPR wt/-* may explain (1) heterogenous findings regarding phenotype as observed in this review (2) lack of severe phenotype in *LEP/LEPR wt/-* in most cases. The question, why humans present *LEP/LEPR wt/-* variants and why heterozygosity does not seem to severely impair carriers is challenging. In general, effects of heterozygosity for specific alleles may be described in an evolutionary perspective as disadvantageous or advantageous. It is conceivable that heterozygosity for *LEP/LEPR* may confer phenotypic advantage in certain environments. Saeed et al. commented on the high prevalence of *LEP* variant p.G133Vfs*15 in Pakistan and discussed this variant as a “founder mutation,” suggesting a possible “heterozygote advantage” [86, 95]. Also for certain *LEPR* mutations detected in Reunion Island, France, a founder effect has been discussed [93]. It must be considered that there could also be a regional reason why these variants offer an advantage there.

*LEP* and *LEPR* heterozygosity seem to confer “thriftiness” to their carriers, as observed in an animal study by Coleman et al. *LEP* and *LEPR* heterozygosity lead to prolonged survival in starvation in mice, due to probable increased metabolic efficiency in heterozygous mice if compared to *wt/wt* [42]. The thrifty gene hypothesis suggests that individuals more prone to store energy or to metabolic efficiency profit during famine times [118]. This hypothesis has been questioned later for being simplistic, but may partly explain recent increase of obesity and diabetes [119]. Evolutionary pressure for efficient energy storage may be the underlying mechanism favoring *LEP/ LEPR wt/-* in certain environments and ethnic groups. Evidence for metabolic differences arising under adipogenic conditions in *LEP/ LEPR wt/-* vs *wt/wt* was provided in several animal studies [40, 49, 51].

### Limitations

Data were found mainly in reports focusing on clinical presentation of *-/-* subjects with *LEP/LEPR* variants, in which *wt/-* relatives were sparely mentioned. This led to a heterogeneous pool of data and scarce information spectrum on human heterozygotes. Moreover, a diagnostic and reporting bias may be underlying. Moreover, we did not compare phenotypes in different ethnic groups. The effect of variants in different genetic backgrounds may lead to a more or less pronounced influence on weight and energy homeostasis and also the effect of a different environment (more or less obesogenic) on the carriers of different variants may lead to an influence on weight and energy homeostasis. Furthermore, the pathogenic degree may vary among variants reported, but no sufficient data on functional studies were available. Effects of *LEP/LEPR* heterozygosity on other organ systems should be investigated, since studies on homozygous phenotype observed leptin influence on lung development [45, 120], bone architecture [47, 92], and cognitive performances [121, 122].

## Conclusion

In this systematic review, we found indications for higher weight status in animals and humans heterozygous for leptin and leptin receptor rare variants. Evidence pointing to higher body fat and differences in leptin levels was found in animal studies only, which supports the assumption of intermediate effects in *LEP/LEPR* heterozygosity. We suggest that *LEP/LEPR* heterozygosity may dispose to overweight and obesity particularly in adulthood, and together with obesogenic factors, it may contribute to the development of obesity. In the context of multifactorial and polygenic obesity, *LEP/LEPR* heterozygosity may represent an underdiagnosed factor. Based on this fresh look on *LEP*/*LEPR* heterozygosity, sophisticated clinical investigations in larger cohorts of *LEP/LEPR* heterozygote humans compared to *wt/wt* relatives and to *wt/wt* population should be considered. Weight status, body fat percentage, leptin levels, metabolic screening for dyslipidemia, glucose intolerance, and fatty liver should be investigated in these cohorts with standardized methods. Results of these studies will enable a deeper understanding of leptin physiology and possible therapeutic applications as well as prevention of obesity and metabolic disease associated with leptin and leptin receptor heterozygosity or partial leptin deficiency.

## Supplementary Information

**Additional file 1:Table S1. **Phenotype of mono-allelic likely pathogenic variants of the leptin (Lep)  gene and the leptin receptor gene (Lepr) in animal models. Table S1A. Phenotype (weight status, body fat, leptin levels, metabolic parameters) of mono-allelic likely pathogenic variants of the leptin gene (*Lep wt/-)* in comparison to wildtype homozygosity (*Lep wt/wt)* phenotype in animal models. Table S1B. Phenotype (weight status, body fat, leptin levels, metabolic parameters) of mono-allelic likely pathogenic variants of the leptin receptor gene (*Lepr wt/-*) in comparison to wildtype homozygosity (*Lepr wt/wt)* phenotype in animal models.

**Additional file 2:Table S2.** Phenotype (body mass index, body fat, leptin levels and metabolic abnormalities) of human mono-allelic likely pathogenic variants of the leptin gene (*LEP wt/-*) in comparison to biallelic likely pathogenic variant carriers (*LEP -/-*), wild type controls (*LEP wt/wt*) and control groups. Differences between *LEP wt/-* and *LEP wt/wt* subjects were summarized in the right columns. References [123–130] details are found in Table S2 and Table S3.

**Additional file 3:Table S3.** Phenotype (body mass index, body fat, leptin levels and metabolic abnormalities) of human mono-allelic likely pathogenic variants of the leptin receptor gene (*LEPR wt/-*) in comparison to biallelic likely pathogenic variant carriers (*LEPR -/-*), wild type controls (*LEPR wt/wt*) and control groups. Differences between *LEPR wt/-* and *LEPR wt/wt* subjects were summarized in the right columns. References [123–130] details are found in Table S2 and Table S3.

## Data Availability

All data analyzed within this review are included in this published article [and its supplementary information files].

## Abbreviations

-/-: biallelic likely pathogenic variant
A: Alanine
BF: Body fat
BMI: Body mass index
CG: Control group
CNS: Central nervous system
G: Guanin
G: Glycine
GH: Growth hormone
GHR: Growth hormone receptor
LEP: Leptin
LEPR: Leptin receptor
MC4R: Melanocortin-4-receptor
PCSK1: Partial prohormone convertase 1
T: Threonine
WHO: World Health Organization
WT: Wild-type homozygosity
wt/-: Mono-allelic likely pathogenetic variant
wt/wt: Wild-type homozygosity

## References

[1] Friedman J (2014). 20 years of leptin: leptin at 20: an overview. J Endocrinol.

[2] Oswal A, Yeo GS (2007). The leptin melanocortin pathway and the control of body weight: lessons from human and murine genetics. Obes Rev.

[3] Farooqi IS, O'Rahilly S (2014). 20 years of leptin: human disorders of leptin action. J Endocrinol.

[4] Ingalls AM, Dickie MM, Snell GD (1950). Obese, a new mutation in the house mouse. J Hered.

[5] Zhang Y, Proenca R, Maffei M, Barone M, Leopold L, Friedman JM (1994). Positional cloning of the mouse obese gene and its human homologue. Nature.

[6] Halaas JL, Gajiwala KS, Maffei M, Cohen SL, Chait BT, Rabinowitz D (1995). Weight-reducing effects of the plasma protein encoded by the obese gene. Science.

[7] Hummel KP, Dickie MM, Coleman DL (1966). Diabetes, a new mutation in the mouse. Science.

[8] Chen H, Charlat O, Tartaglia LA, Woolf EA, Weng X, Ellis SJ (1996). Evidence that the diabetes gene encodes the leptin receptor: identification of a mutation in the leptin receptor gene in db/db mice. Cell.

[9] Lee GH, Proenca R, Montez JM, Carroll KM, Darvishzadeh JG, Lee JI (1996). Abnormal splicing of the leptin receptor in diabetic mice. Nature.

[10] Truett GE, Bahary N, Friedman JM, Leibel RL (1991). Rat obesity gene fatty (fa) maps to chromosome 5: evidence for homology with the mouse gene diabetes (db). Proc Natl Acad Sci U S A.

[11] Coleman DL (1973). Effects of parabiosis of obese with diabetes and normal mice. Diabetologia.

[12] Coleman DL, Hummel KP (1969). Effects of parabiosis of normal with genetically diabetic mice. Am J Physiol.

[13] Campfield LA, Smith FJ, Guisez Y, Devos R, Burn P (1995). Recombinant mouse OB protein: evidence for a peripheral signal linking adiposity and central neural networks. Science.

[14] Stephens TW, Basinski M, Bristow PK, Bue-Valleskey JM, Burgett SG, Craft L (1995). The role of neuropeptide Y in the antiobesity action of the obese gene product. Nature.

[15] Barrenetxe J, Villaro AC, Guembe L, Pascual I, Munoz-Navas M, Barber A (2002). Distribution of the long leptin receptor isoform in brush border, basolateral membrane, and cytoplasm of enterocytes. Gut.

[16] Huynh FK, Neumann UH, Wang Y, Rodrigues B, Kieffer TJ, Covey SD (2013). A role for hepatic leptin signaling in lipid metabolism via altered very low density lipoprotein composition and liver lipase activity in mice. Hepatology.

[17] Solberg R, Aas V, Thoresen GH, Kase ET, Drevon CA, Rustan AC (2005). Leptin expression in human primary skeletal muscle cells is reduced during differentiation. J Cell Biochem.

[18] Soll AH, Kahn CR, Neville DM (1975). Insulin binding to liver plasm membranes in the obese hyperglycemic (ob/ob) mouse. Demonstration of a decreased number of functionally normal receptors. J Biol Chem.

[19] Montague CT, Farooqi IS, Whitehead JP, Soos MA, Rau H, Wareham NJ (1997). Congenital leptin deficiency is associated with severe early-onset obesity in humans. Nature.

[20] Clement K, Vaisse C, Lahlou N, Cabrol S, Pelloux V, Cassuto D (1998). A mutation in the human leptin receptor gene causes obesity and pituitary dysfunction. Nature.

[21] Pigeyre M, Yazdi FT, Kaur Y, Meyre D (2016). Recent progress in genetics, epigenetics and metagenomics unveils the pathophysiology of human obesity. Clin Sci (Lond).

[22] Strobel A, Issad T, Camoin L, Ozata M, Strosberg AD (1998). A leptin missense mutation associated with hypogonadism and morbid obesity. Nat Genet.

[23] Funcke JB, von Schnurbein J, Lennerz B, Lahr G, Debatin KM, Fischer-Posovszky P (2014). Monogenic forms of childhood obesity due to mutations in the leptin gene. Mol Cell Pediatr.

[24] Huvenne H, Dubern B, Clement K, Poitou C (2016). Rare genetic forms of obesity: clinical approach and current treatments in 2016. Obes Facts.

[25] Nunziata A, Funcke JB, Borck G, von Schnurbein J, Brandt S, Lennerz B (2019). Functional and phenotypic characteristics of human leptin receptor mutations. J Endocr Soc.

[26] Nunziata A, Borck G, Funcke JB, Kohlsdorf K, Brandt S, Hinney A (2017). Estimated prevalence of potentially damaging variants in the leptin gene. Mol Cell Pediatr.

[27] Ayers KL, Glicksberg BS, Garfield AS, Longerich S, White JA, Yang P (2018). Melanocortin 4 receptor pathway dysfunction in obesity: patient stratification aimed at MC4R agonist treatment. J Clin Endocrinol Metab.

[28] Vogel F (1984). Clinical consequences of heterozygosity for autosomal-recessive diseases. Clin Genet.

[29] Blum WF, Klammt J, Amselem S, Pfaffle HM, Legendre M, Sobrier ML (2018). Screening a large pediatric cohort with GH deficiency for mutations in genes regulating pituitary development and GH secretion: frequencies, phenotypes and growth outcomes. EBioMedicine.

[30] Porto WF, Marques FA, Pogue HB, de Oliveira Cardoso MT, do Vale MGR, da Silva Pires A (2017). Computational investigation of growth hormone receptor Trp169Arg heterozygous mutation in a child with short stature. J Cell Biochem.

[31] Konno T, Tada M, Tada M, Koyama A, Nozaki H, Harigaya Y (2014). Haploinsufficiency of CSF-1R and clinicopathologic characterization in patients with HDLS. Neurology.

[32] Farooqi IS, Keogh JM, Kamath S, Jones S, Gibson WT, Trussell R (2001). Partial leptin deficiency and human adiposity. Nature.

[33] Shabana, Hasnain S (2016). The p. N103K mutation of leptin (LEP) gene and severe early onset obesity in Pakistan. Biol Res.

[34] Group WHOMGRS (2006). WHO child growth standards based on length/height, weight and age. Acta Paediatr Suppl..

[35] Wabitsch M, Pridzun L, Ranke M, von Schnurbein J, Moss A, Brandt S (2017). Measurement of immunofunctional leptin to detect and monitor patients with functional leptin deficiency. Eur J Endocrinol.

[36] Lahlou N, Clement K, Carel JC, Vaisse C, Lotton C, Le Bihan Y (2000). Soluble leptin receptor in serum of subjects with complete resistance to leptin: relation to fat mass. Diabetes.

[37] Lahlou N, Issad T, Lebouc Y, Carel JC, Camoin L, Roger M (2002). Mutations in the human leptin and leptin receptor genes as models of serum leptin receptor regulation. Diabetes.

[38] Karvonen MK, Pesonen U, Heinonen P, Laakso M, Rissanen A, Naukkarinen H (1998). Identification of new sequence variants in the leptin gene. J Clin Endocrinol Metab.

[39] den Dunnen JT, Dalgleish R, Maglott DR, Hart RK, Greenblatt MS, McGowan-Jordan J (2016). HGVS recommendations for the description of sequence variants: 2016 update. Hum Mutat.

[40] Begriche K, Letteron P, Abbey-Toby A, Vadrot N, Robin MA, Bado A (2008). Partial leptin deficiency favors diet-induced obesity and related metabolic disorders in mice. Am J Physiol Endocrinol Metab.

[41] Chung WK, Belfi K, Chua M, Wiley J, Mackintosh R, Nicolson M (1998). Heterozygosity for Lep(ob) or Lep(rdb) affects body composition and leptin homeostasis in adult mice. Am J Physiol.

[42] Coleman DL (1979). Obesity genes: beneficial effects in heterozygous mice. Science.

[43] Flatt PR, Bailey CJ (1981). Abnormal plasma glucose and insulin responses in heterozygous lean (ob/+) mice. Diabetologia.

[44] Haller EW, Wittmers LE, Haller IV, Regal RR (1999). The obese gene is expressed in lean littermates of the genetically obese mouse (C57BL/6J ob/ob). Am J Physiol.

[45] Huang K, Rabold R, Abston E, Schofield B, Misra V, Galdzicka E (2008). Effects of leptin deficiency on postnatal lung development in mice. J Appl Physiol (1985).

[46] Lee VK, Hosking BM, Holeniewska J, Kubala EC, Lundh von Leithner P, Gardner PJ (2018). BTBR ob/ob mouse model of type 2 diabetes exhibits early loss of retinal function and retinal inflammation followed by late vascular changes. Diabetologia.

[47] Philbrick KA, Turner RT, Branscum AJ, Wong CP, Iwaniec UT (2015). Paradoxical effects of partial leptin deficiency on bone in growing female mice. Anat Rec (Hoboken).

[48] Sena A, Rebel G, Bieth R, Hubert P, Waksman A (1982). Lipid composition in liver and brain of genetically obese (ob/ob), heterozygote (ob/+)and normal (+/+) mice. Biochim Biophys Acta.

[49] Swartz-Basile DA, Goldblatt MI, Choi SH, Svatek C, Tran K, Nakeeb A (2006). Biliary lipids and cholesterol crystal formation in leptin-deficient obese mice. HPB (Oxford).

[50] Tran KQ, Goldblatt MI, Swartz-Basile DA, Svatek C, Nakeeb A, Pitt HA (2003). Diabetes and hyperlipidemia correlate with gallbladder contractility in leptin-related murine obesity. J Gastrointest Surg.

[51] Trevaskis JL, Meyer EA, Galgani JE, Butler AA (2008). Counterintuitive effects of double-heterozygous null melanocortin-4 receptor and leptin genes on diet-induced obesity and insulin resistance in C57BL/6J mice. Endocrinology.

[52] Yen TT, Lowry L, Steinmetz J (1968). Obese locus in Mus musculus: a gene dosage effect. Biochem Biophys Res Commun.

[53] Chebel RC, Susca F, Santos JE (2008). Leptin genotype is associated with lactation performance and health of Holstein cows. J Dairy Sci.

[54] Choi HM, Kim HR, Kim EK, Byun YS, Won YS, Yoon WK (2015). An age-dependent alteration of the respiratory exchange ratio in the db/db mouse. Lab Anim Res.

[55] Harrod JS, Rada CC, Pierce SL, England SK, Lamping KG (2011). Altered contribution of RhoA/Rho kinase signaling in contractile activity of myometrium in leptin receptor-deficient mice. Am J Physiol Endocrinol Metab.

[56] Hirose Y, Hata K, Kuno T, Yoshida K, Sakata K, Yamada Y (2004). Enhancement of development of azoxymethane-induced colonic premalignant lesions in C57BL/KsJ-db/db mice. Carcinogenesis.

[57] Kanda Y, Shimoda M, Tawaramoto K, Hamamoto S, Tatsumi F, Kawasaki F (2009). Molecular analysis of db gene-related pancreatic beta cell dysfunction; evidence for a compensatory mechanism inhibiting development of diabetes in the db gene heterozygote. Endocr J.

[58] Levine DZ, Iacovitti M, Robertson SJ, Mokhtar GA (2006). Modulation of single-nephron GFR in the db/db mouse model of type 2 diabetes mellitus. Am J Physiol Regul Integr Comp Physiol.

[59] Nadif R, Dilworth MR, Sibley CP, Baker PN, Davidge ST, Gibson JM (2015). The maternal environment programs postnatal weight gain and glucose tolerance of male offspring, but placental and fetal growth are determined by fetal genotype in the Leprdb/+ model of gestational diabetes. Endocrinology.

[60] Plows JF, Yu X, Broadhurst R, Vickers MH, Tong C, Zhang H (2017). Absence of a gestational diabetes phenotype in the LepRdb/+ mouse is independent of control strain, diet, misty allele, or parity. Sci Rep.

[61] Pollock KE, Stevens D, Pennington KA, Thaisrivongs R, Kaiser J, Ellersieck MR (2015). Hyperleptinemia during pregnancy decreases adult weight of offspring and is associated with increased offspring locomotor activity in mice. Endocrinology.

[62] Shi H, Patschan D, Epstein T, Goligorsky MS, Winaver J (2007). Delayed recovery of renal regional blood flow in diabetic mice subjected to acute ischemic kidney injury. Am J Physiol Renal Physiol.

[63] Stanley JL, Cheung CC, Rueda-Clausen CF, Sankaralingam S, Baker PN, Davidge ST (2011). Effect of gestational diabetes on maternal artery function. Reprod Sci.

[64] Yamashita H, Shao J, Ishizuka T, Klepcyk PJ, Muhlenkamp P, Qiao L (2001). Leptin administration prevents spontaneous gestational diabetes in heterozygous Lepr(db/+) mice: effects on placental leptin and fetal growth. Endocrinology.

[65] Yamashita H, Shao J, Qiao L, Pagliassotti M, Friedman JE (2003). Effect of spontaneous gestational diabetes on fetal and postnatal hepatic insulin resistance in Lepr(db/+) mice. Pediatr Res.

[66] Blonz ER, Stern JS, Curry DL (1985). Dynamics of pancreatic insulin release in young Zucker rats: a heterozygote effect. Am J Physiol.

[67] Cleary MP, Phillips FC (1999). The presence of the “fa” gene in heterozygous (FA/fa) lean female rats, effects on body weight, body fat and serum leptin. Obes Res.

[68] Heo YR, Claycombe K, Jones BH, Wright P, Truett GE, Zemel M (2002). Effects of fatty (fa) allele and high-fat diet on adipose tissue leptin and lipid metabolism. Horm Metab Res.

[69] Himeno K, Seike M, Fukuchi S, Masaki T, Kakuma T, Sakata T (2009). Heterozygosity for leptin receptor (fa) accelerates hepatic triglyceride accumulation without hyperphagia in Zucker rats. Obes Res Clin Pract.

[70] Kowalski TJ, Ster AM, Smith GP (1998). Ontogeny of hyperphagia in the Zucker (fa/fa) rat. Am J Physiol.

[71] Kraeft S, Schwarzer K, Eiden S, Nuesslein-Hildesheim B, Preibisch G, Schmidt I (1999). Leptin responsiveness and gene dosage for leptin receptor mutation (fa) in newborn rats. Am J Physiol.

[72] Masuyama T, Katsuda Y, Shinohara M (2005). A novel model of obesity-related diabetes: introgression of the Lepr(fa) allele of the Zucker fatty rat into nonobese Spontaneously Diabetic Torii (SDT) rats. Exp Anim.

[73] Moralejo DH, Hansen CT, Treuting P, Hessner MJ, Fuller JM, Van Yserloo B (2010). Differential effects of leptin receptor mutation on male and female BBDR Gimap5-/Gimap5- spontaneously diabetic rats. Physiol Genomics.

[74] Phillips FC, Cleary MP (1994). Metabolic measurements among homozygous (fa/fa) obese, heterozygous (Fa/fa) lean and homozygous (Fa/Fa) lean Zucker rat pups at 17 days of age. J Nutr.

[75] Schwarzer K, Doring H, Schmidt I (1997). Different physiological traits underlying increased body fat of fatty (fa/fa) and heterozygous (+/fa) rats. Am J Physiol.

[76] Tamasi JA, Arey BJ, Bertolini DR, Feyen JH (2003). Characterization of bone structure in leptin receptor-deficient Zucker (fa/fa) rats. J Bone Miner Res.

[77] York D, Holt S, Rothwell N, Stock M (1984). Effect of age and gene dosage on brown adipose tissue of Zucker obese fa/fa rats. Am J Physiol.

[78] Zhang Y, Olbort M, Schwarzer K, Nuesslein-Hildesheim B, Nicolson M, Murphy E (1997). The leptin receptor mediates apparent autocrine regulation of leptin gene expression. Biochem Biophys Res Commun.

[79] Haldar A, French MC, Brauning R, Edwards SJ, O'Connell AR, Farquhar PA (2014). Single-nucleotide polymorphisms in the LEPR gene are associated with divergent phenotypes for age at onset of puberty in Davisdale ewes. Biol Reprod.

[80] Mantzoros CS, Ozata M, Negrao AB, Suchard MA, Ziotopoulou M, Caglayan S (2001). Synchronicity of frequently sampled thyrotropin (TSH) and leptin concentrations in healthy adults and leptin-deficient subjects: evidence for possible partial TSH regulation by leptin in humans. J Clin Endocrinol Metab.

[81] Murray PG, Read A, Banerjee I, Whatmore AJ, Pritchard LE, Davies RA (2011). Reduced appetite and body mass index with delayed puberty in a mother and son: association with a rare novel sequence variant in the leptin gene. Eur J Endocrinol.

[82] Ozata M, Ozdemir IC, Licinio J (1999). Human leptin deficiency caused by a missense mutation: multiple endocrine defects, decreased sympathetic tone, and immune system dysfunction indicate new targets for leptin action, greater central than peripheral resistance to the effects of leptin, and spontaneous correction of leptin-mediated defects. J Clin Endocrinol Metab.

[83] Yupanqui-Lozno H, Bastarrachea RA, Yupanqui-Velazco ME, Alvarez-Jaramillo M, Medina-Mendez E, Giraldo-Pena AP, et al. Congenital leptin deficiency and leptin gene missense mutation found in two colombian sisters with severe obesity. Genes (Basel). 2019;10(5). 10.3390/genes10050342. 10.3390/genes10050342 PMC6562380 31067764

[84] Wabitsch M, Funcke JB, von Schnurbein J, Denzer F, Lahr G, Mazen I (2015). Severe early-onset obesity due to bioinactive leptin caused by a p.N103K Mutation in the Leptin Gene. J Clin Endocrinol Metab.

[85] Ozata M, Avcu F, Durmus O, Yilmaz I, Ozdemir IC, Yalcin A (2001). Leptin does not play a major role in platelet aggregation in obesity and leptin deficiency. Obes Res.

[86] Saeed S, Butt TA, Anwer M, Arslan M, Froguel P (2012). High prevalence of leptin and melanocortin-4 receptor gene mutations in children with severe obesity from Pakistani consanguineous families. Mol Genet Metab.

[87] Ozata M, Uckaya G, Aydin A, Isimer A, Ozdemir IC (2000). Defective antioxidant defense system in patients with a human leptin gene mutation. Horm Metab Res.

[88] Saeed S, Bech PR, Hafeez T, Alam R, Falchi M, Ghatei MA (2014). Changes in levels of peripheral hormones controlling appetite are inconsistent with hyperphagia in leptin-deficient subjects. Endocrine.

[89] Zhao Y, Hong N, Liu X, Wu B, Tang S, Yang J (2014). A novel mutation in leptin gene is associated with severe obesity in Chinese individuals. Biomed Res Int.

[90] Fatima W, Shahid A, Imran M, Manzoor J, Hasnain S, Rana S (2011). Leptin deficiency and leptin gene mutations in obese children from Pakistan. Int J Pediatr Obes.

[91] Farooqi IS, Wangensteen T, Collins S, Kimber W, Matarese G, Keogh JM (2007). Clinical and molecular genetic spectrum of congenital deficiency of the leptin receptor. N Engl J Med.

[92] Hannema SE, Wit JM, Houdijk ME, van Haeringen A, Bik EC, Verkerk AJ (2016). Novel leptin receptor mutations identified in two girls with severe obesity are associated with increased bone mineral density. Horm Res Paediatr.

[93] Huvenne H, Le Beyec J, Pepin D, Alili R, Kherchiche PP, Jeannic E (2015). Seven novel deleterious LEPR mutations found in early-onset obesity: a DeltaExon6-8 shared by subjects from Reunion Island, France, suggests a founder effect. J Clin Endocrinol Metab.

[94] Saeed S, Bonnefond A, Manzoor J, Philippe J, Durand E, Arshad M (2014). Novel LEPR mutations in obese Pakistani children identified by PCR-based enrichment and next generation sequencing. Obesity (Silver Spring).

[95] Saeed S, Bonnefond A, Manzoor J, Shabbir F, Ayesha H, Philippe J (2015). Genetic variants in LEP, LEPR, and MC4R explain 30% of severe obesity in children from a consanguineous population. Obesity (Silver Spring).

[96] Nordang GBN, Busk OL, Tveten K, Hanevik HI, Fell AKM, Hjelmesaeth J (2017). Next-generation sequencing of the monogenic obesity genes LEP, LEPR, MC4R, PCSK1 and POMC in a Norwegian cohort of patients with morbid obesity and normal weight controls. Mol Genet Metab.

[97] Voigtmann F, Wolf P, Landgraf K, Stein R, Kratzsch J, Schmitz S (2021). Identification of a novel leptin receptor (LEPR) variant and proof of functional relevance directing treatment decisions in patients with morbid obesity. Metabolism.

[98] Lotta LA, Mokrosinski J, Mendes de Oliveira E, Li C, Sharp SJ, Luan J (2019). Human gain-of-function MC4R variants show signaling bias and protect against obesity. Cell.

[99] Stutzmann F, Tan K, Vatin V, Dina C, Jouret B, Tichet J (2008). Prevalence of melanocortin-4 receptor deficiency in Europeans and their age-dependent penetrance in multigenerational pedigrees. Diabetes.

[100] Echwald SM, Rasmussen SB, Sorensen TI, Andersen T, Tybjaerg-Hansen A, Clausen JO (1997). Identification of two novel missense mutations in the human OB gene. Int J Obes Relat Metab Disord.

[101] Maher MA, Banz WJ, Truett GE, Zemel MB (1996). Dietary fat and sex modify heterozygote effects of the rat fatty (fa) allele. J Nutr.

[102] Truett GE, Tempelman RJ, Walker JA (1995). Codominant effects of the fatty (fa) gene during early development of obesity. Am J Physiol.

[103] Ahima RS, Prabakaran D, Mantzoros C, Qu D, Lowell B, Maratos-Flier E (1996). Role of leptin in the neuroendocrine response to fasting. Nature.

[104] Flier JS (1998). Clinical review 94: what’s in a name? In search of leptin&apos;s physiologic role. J Clin Endocrinol Metab.

[105] Flier JS, Maratos-Flier E (2017). Leptin’s physiologic role: does the emperor of energy balance have no clothes?. Cell Metab.

[106] Ravussin E, Pratley RE, Maffei M, Wang H, Friedman JM, Bennett PH (1997). Relatively low plasma leptin concentrations precede weight gain in Pima Indians. Nat Med.

[107] Clement K, Biebermann H, Farooqi IS, Van der Ploeg L, Wolters B, Poitou C (2018). MC4R agonism promotes durable weight loss in patients with leptin receptor deficiency. Nat Med.

[108] Leibel RL (1997). And finally, genes for human obesity. Nat Genet.

[109] Potoczna N, Branson R, Kral JG, Piec G, Steffen R, Ricklin T (2004). Gene variants and binge eating as predictors of comorbidity and outcome of treatment in severe obesity. J Gastrointest Surg.

[110] Wilkie AO (1994). The molecular basis of genetic dominance. J Med Genet.

[111] Drabkin M, Birk OS, Birk R (2018). Heterozygous versus homozygous phenotype caused by the same MC4R mutation: novel mutation affecting a large consanguineous kindred. BMC Med Genet.

[112] Peelman F, Zabeau L, Moharana K, Savvides SN, Tavernier J (2014). 20 years of leptin: insights into signaling assemblies of the leptin receptor. J Endocrinol.

[113] Ward AC, van Aesch YM, Gits J, Schelen AM, de Koning JP, van Leeuwen D (1999). Novel point mutation in the extracellular domain of the granulocyte colony-stimulating factor (G-CSF) receptor in a case of severe congenital neutropenia hyporesponsive to G-CSF treatment. J Exp Med.

[114] Sinha S, Zhu QS, Romero G, Corey SJ (2003). Deletional mutation of the external domain of the human granulocyte colony-stimulating factor receptor in a patient with severe chronic neutropenia refractory to granulocyte colony-stimulating factor. J Pediatr Hematol Oncol.

[115] Creemers JW, Choquet H, Stijnen P, Vatin V, Pigeyre M, Beckers S (2012). Heterozygous mutations causing partial prohormone convertase 1 deficiency contribute to human obesity. Diabetes.

[116] Stijnen P, Ramos-Molina B, O'Rahilly S, Creemers JW (2016). PCSK1 Mutations and human endocrinopathies: from obesity to gastrointestinal disorders. Endocr Rev.

[117] Zabeau L, Jensen CJ, Seeuws S, Venken K, Verhee A, Catteeuw D (2015). Leptin’s metabolic and immune functions can be uncoupled at the ligand/receptor interaction level. Cell Mol Life Sci.

[118] Neel JV (1962). Diabetes mellitus: a “thrifty” genotype rendered detrimental by “progress”?. Am J Hum Genet.

[119] Reddon H, Patel Y, Turcotte M, Pigeyre M, Meyre D (2018). Revisiting the evolutionary origins of obesity: lazy versus peppy-thrifty genotype hypothesis. Obes Rev.

[120] Lawrence S, Warshaw J, Nielsen HC (1989). Delayed lung maturation in the macrosomic offspring of genetically determined diabetic (db/+) mice. Pediatr Res.

[121] Paz-Filho GJ, Babikian T, Asarnow R, Delibasi T, Esposito K, Erol HK (2008). Leptin replacement improves cognitive development. PLoS ONE.

[122] Shanley LJ, Irving AJ, Harvey J (2001). Leptin enhances NMDA receptor function and modulates hippocampal synaptic plasticity. J Neurosci.

[123] Mazen I, El-Gammal M, Abdel-Hamid M, Amr K (2009). A novel homozygous missense mutation of the leptin gene (N103K) in an obese Egyptian patient. Mol Genet Metab.

[124] Fischer-Posovszky P, von Schnurbein J, Moepps B, Lahr G, Strauss G, Barth TF, Kassubek J, Mühleder H, Möller P, Debatin KM, Gierschik P, Wabitsch P (2010). A new missense mutation in the leptin gene causes mild obesity and hypogonadism without affecting T cell responsiveness. J Clin Endocrinol Metab.

[125] Thakur S, Kumar A, Dubey S, Saxena R, Peters ANC, Singhal A (2014). A novel mutation of the leptin gene in an Indian patient. Clin Genet.

[126] Dayal D, Seetharaman K, Panigrahi I, Muthuvel B, Agarwal A (2018). Severe early onset obesity due to a novel missense mutation in Exon 3 of the leptin gene in an infant from Northwest India. J Clin Res Pediatr Endocrinol.

[127] Branson R, Potoczna N, Kral John G, Klaus-Ulrich L, Hoehe Margret R, Horber Fritz F (2003). Binge eating as a major phenotype of melanocortin 4 receptor gene mutations 2003. N Engl J Med.

[128] Mazen I, El-Gammal M, Abdel-Hamid M, Farooqi IS, Amr K (2011). Homozygosity for a novel missense mutation in the leptin receptor gene (P316T) in two Egyptian cousins with severe early onset obesity. Mol Genet Metab.

[129] Dehghani MR, Mehrjardi MYV, Dilaver N, Tajamolian M, Enayati S, Ebrahimi P (2018). Potential role of gender specific effect of leptin receptor deficiency in an extended consanguineous family with severe early-onset obesity. Eur J Med Genet.

[130] Akıncı A, Türkkahraman D, Tekedereli I, Özer L, Evren B, Şahin I, Kalkan T, Çürek Y, Çamtosun E, Döğer E, Bideci A, Güven A, Eren E, Sangün Ö, Çayır A, Bilir P, Törel Ergür A, Ercan O (2019). Novel mutations in obesity-related genes in turkish children with non-syndromic early onset severe obesity: a multicentre study. J Clin Res Pediatr Endocrinol.

