# Supplementary material for: A fresh look to the phenotype in mono-allelic likely pathogenic variants of the leptin and the leptin receptor gene
Source: Mol Cell Pediatr. 2021 Aug 26;8:10. doi: 10.1186/s40348-021-00119-7 (PMC8390564; doi:10.1186/s40348-021-00119-7)
Supplement: Supplementary file 1 — Additional file 1:Table S1. Phenotype of mono-allelic likely pathogenic variants of the leptin (Lep) gene and the leptin receptor gene (Lepr) in animal models. Table S1A. Phenotype (weight status, body fat, leptin levels, metabolic parameters) of mono-allelic likely pathogenic variants of the leptin gene (Lep wt/-) in comparison to wildtype homozygosity (Lep wt/wt) phenotype in animal models. Table S1B. Phenotype (weight status, body fat, leptin levels, metabolic parameters) of mono-allelic likely pathogenic variants of the leptin receptor gene (Lepr wt/-) in comparison to wildtype homozygosity (Lepr wt/wt) phenotype in animal models. [file 40348_2021_119_MOESM1_ESM.docx]

**Table S1**. Phenotype of mono-allelic likely pathogenic variants of the leptin (*Lep*) and the leptin receptor gene (*Lepr*) in animal models.

**Table S1A**. Phenotype (weight status, body fat, leptin levels and metabolic parameters) of mono-allelic likely pathogenic variants of the leptin gene (*Lep wt/-*) in comparison to wildtype homozygosity (*Lep wt/wt*) phenotype in animal models.

| **Author, year** | **Animal model** | **Weight status** | **Fat mass** | **Circulating leptin concentrations** | **Metabolic abnormalities** | **Differences *Lep* wt/- vs wt/wt:** **Weight status (W), Leptin (L), Metabolic (M)** |
| --- | --- | --- | --- | --- | --- | --- |
| Yen et al. 1968(52) | mouse; C57BL/6J *wt/wt* vs *Lep* wt/- vs *Lep* -/-, 3-5 months age | *Lep* wt/- had similar body weight to *wt/wt* | n.a. | n.a. | Similar blood glucose to *wt/wt*; *Lep* wt/- showed intermediate CO2 oxidation in fat tissue (between values of *wt/wt* and *-/-*) | W-, M- |
| Soll et al. 1975(18) | mouse; C57BL/6J *wt/wt* vs *Lep* wt/- vs *Lep* -/-, thin littermates of equal age | *Lep* wt/- had similar body weight to thin littermates and *wt/wt* | n.a. | n.a. | Similar plasma glucose, plasma insulin and insulin binding to receptors in *Le p*wt/- and WT | W-, M- |
| Coleman 1979(42) | mouse; male C57BL/6J and C57BL/KsJ *wt/wt* vs *Lep* wt/-, 7-9 months of age; chow diet, total fast | No differences between *Lep* wt/- and *wt/wt* in starting weight | n.a. | n.a. | No differences between *Lep* wt/- and *wt/wt* in plasma insulin, blood sugar or liver glycogen concentration; differences in survival time after total fast (*Le p*wt/- showed longer survival time) | W-, M- |
| Flatt and Bailey 1981(43) | mouse; C57BL/6J outcrossed to JH, CRL and conseq.to local strains (Aston stock) wt/wt, *Lep* wt/-, *Lep*-/-, males, at 20 weeks of age | No differences in body weight between *wt/wt* and *Lep* wt/- | n.a. | n.a. | Plasma glucose and insulin concentrations were higher in *Lep* wt/- than *wt/wt* mice (p<0.05) | W-, M+ |
| Sena et al. 1982(48) | mouse; C57BL/6J  *wt/wt*, *Lep* wt/-, *Lep*-/- at 5-7 months age | Brain weight about 35% lower in *Lep* wt/-and *Lep*+/+ than in  *wt/wt* (p<0.05); liver weight in *Lep* wt/- similar to liver weight in  *wt/wt* | Lower cholesterol and phospholipid weight in brain and liver *Lep* wt/- vs  *wt/wt* (p<0.05); ratio unsaturated/saturated FA lower in *Lep* wt/- than in  *wt/wt* in liver (p<0.05), higher than in  *wt/wt* in brain (p<0.05); higher fatty acid melting point of liver in *Lep*wt/-vs  *wt/wt*(p<0.05) | n.a. | n.a. |  |
| Chung et al. 1998(41) | mouse; C57BL/6J *Lep* wt/- and  *wt/wt*; sacrificed between 39 and 120 days after 2-h fast; observational study | no difference in BMI between *Lep* wt/- vs  *wt/wt* | 26.7% higher in *Lep* wt/- than in  *wt/wt* (p<0.05), BF% (adjusted for age and sex) 23.5% higher in *Lep* wt/-than in  *wt/wt* (p<0.05) | Mean levels comparable between *Lep* wt/- and  *wt/wt*; if adjusted for fat mass, 32.8% lower in *Lep* wt/- than in  *wt/wt* (p<0.05) | n.a. | W+, L- |
| Haller et al. 1999(44) | mouse; C57BL/6J  *wt/wt*, *Lep*wt/- , *Lep*-/- at comparable ages (62-364 days) | Female *Lep* wt/- were heavier than female  *wt/wt* (p<0.05); male *Lep* wt/- were not heavier than male  *wt/wt* (p>0.05) | n.a. | n.a. | Fasting blood glucose was higher in female *Lep*wt/- than in female  *wt/wt* (p<0.05); it was not different between male *Lep*wt/- and male  *wt/wt* (p>0,05) | W+, M+ |
| Tran et al. 2003(50) | mouse; C57BL/6J females;  *wt/wt*, *Lep* wt/-, *Lep* -/- (chow diet) | Slightly higher body weight at 8 weeks of in *Lep* wt/- than in  *wt/wt* animals (p<0.05) | n.a. | n.a. | No differences in insulin, glucose, and cholesterol between *Lep* wt/- and  *wt/wt*; TG levels higher in *Lep* wt/- than in  *wt/wt* (p<0.05) | W+, M+ |
| Swartz-Basile et al. 2006(49) | mouse; C57Bl/6J lean  *wt/wt* females, *Lep* wt/- and *Lep* -/- ; 12 weeks age (chow or lithogenic diet) | *Lep*wt/- had slightly higher body weight compared to  *wt/wt* in both diets (p<0.05) | n.a. | Leptin levels lower in *Lep* wt/- vs  *wt/wt* in both diets (p<0.05) | Lithogenic diet increased serum cholesterol, biliary cholesterol crystals, HDL and serum glucose levels in *Lep* wt/- vs  *wt/wt*; *Lep* wt/- had similar number of hepatic fat vacuoles as  *wt/wt*; | W+, L+, M+ |
| Begriche et al. 2008(40) | mouse; male C57BL/6J  *wt/wt* and C57BL6- *Lep* wt/-, 5-weeks old under SCD or HFD | increased initial body weight in *Lep* wt/- than in  *wt/wt* (p<0.05) | Initial body fat mass significantly increased in *Lep* wt/- compared to  *wt/wt* (p<0.05) | Initial plasma leptin levels similar in *Lep* wt/- and  *wt/wt*; relative leptin level (expressed per gram of fat mass) was lower in *Lep* wt/- compared to  *wt/wt* (p<0.05) | *Lep* wt/- under SCD: slight glucose intolerance, increased plasma levels of ALT vs  *wt/wt*; HFD: triglycerides, total cholesterol and GLP-1 significantly increased in *Lep* wt/- vs  *wt/wt* (p<0.05) | W+, L+, M+ |
| Huang et al. 2008(45) | mouse; C57BL/6J  *wt/wt*, *Lep* wt/- and *Lep*-/- at 2,3,4 and 7 weeks of age | No difference in body weight between *Lep* wt/- and  *wt/wt* | n.a. | n.a. | n.a. | W- |
| Trevaskis et al. 2008(51) | mouse; C57BL/6J:  *wt/wt* vs *Lep*wt/- vs. Dbl-Het vs Mc4rHet; group (1) LFD from weaning until 28 weeks of age; group (2) HFD 17-28 weeks of age | Body weight in *Lep* wt/- was not significantly different from  *wt/wt* in both diets | *Lep* wt/- had greater  FM compared with  *wt/wt* (p<0.05\| only for females) | *Lep* wt/-: disproportionately lower serum leptin as a function of percent FM vs leptin in  *wt/wt* (p<0.05) | Fasting insulin levels in *Lep* wt/- were not significantly different from  *wt/wt* under LFD, fasting insulin levels were significantly different from *wt/wt* under HFD (only in male mice) | W-, L+, M+ |
| Chebel et al. 2008(53) | cow; SNP in the R4C locus in exon 2 of the leptin gene:  *wt/wt*, *Lep* wt/-, *Lep*-/-; examination of differences in milk composition | Slightly lower body weight in heterozygous cows (p<0.05) | n.a. | n.a. | n.a. | W- |
| Philbrick et al. 2015(47) | mouse; female C57BL/6  *wt/wt*, *Lep* wt/-, *Lep*-/- at 7 weeks age | *Lep* wt/- mice had greater body weight than  *wt/wt* (p<0.05) | *Lep* wt/- had greater abdominal WAT weight than  *wt/wt* (p<0.05) | Lower leptin levels in *Lep* wt/- mice than in  *wt/wt* (p<0.05) | n.a. | W+, L+ |
| Lee et al. 2018(46) | mouse; BTBR  *wt/wt*, *Lep* wt/-, *Lep* -/- mice at 6,10, 15 and 20 weeks age | Similar weight in *Lep* wt/- and  *wt/wt* | n.a. | n.a. | Similar blood glucose levels in *Lep* wt/- and  *wt/wt* | W-, M- |

Abbreviations: ALT: alanine aminotransferase; FA: fatty acids; FM: fat mass; HFD: high fat diet; LFD: low fat diet; n.a.: not available; SCD: standard calory diet; SNP: single nucleotide polymorphismus; TG: triglycerides; WAT: white adipose tissue, /: not available. Differences *Lep wt/-* vs wt/wt: weight status and body fat, L: leptin levels, M: metabolic abnormalities. -: no differences observed; +: differences observed.

**Table S1B**. Phenotype (weight status, body fat, leptin levels and metabolic parameters) of mono-allelic likely pathogenic variants of the leptin receptor gene (*Lepr wt/-*) in comparison to wildtype homozygosity (*Lepr wt/wt*) phenotype in animal models.

| **Author, year** | **animal model** | **Weight status** | **Fat mass** | **Circulating leptin concentrations** | **Metabolic abnormalities** | **Differences *Lepr wt/-* vs wt/wt:** **Weight status (W), Leptin (L), Metabolic (M)** |
| --- | --- | --- | --- | --- | --- | --- |
| Coleman 1979(42) | mouse; C57BL/6J and C57BLKS/J wt/wt, *Lepr wt/-*, 7-9 months of age | no differences between wt/wt and *Lepr wt/-* in starting body weight | n.a. | n.a. | No differences between wt/wt and *Lepr wt/-* in plasma insulin, blood sugar or liver glycogen concentration | W-, M- |
| York et al. 1984(77) | Zucker rat; wt/wt vs *Lepr wt/-* vs *Lepr -/-* at 8-10 week age | wt/wt and *Lepr wt/-* were both lean | wt/wt and *Lepr wt/-* showed similar wet weight of brown adipose tissue | n.a. | Significant gene-dosage effect shown for free fatty acids, triiodothyronine; serum insulin levels similar in *Lepr wt/-* vs wt/wt (p<0.05);  gene-dosage effect for GDP binding to interscapular brown adipose tissue mitochondria and for oxygen consumption after a meal or norepinephrine injection | W-, M+ |
| Blonz et al. 1985(66) | Zucker rat; wt/wt vs *Lepr wt/-* and *Lepr -/-* at 2 and 4 weeks age | Significantly higher in *Lepr wt/-* vs wt/wt; carcass protein and fat-free weight significantly higher in *Lepr wt/-* vs wt/wt (p<0.05) | Carcass fat and BF% significantly higher in *Lepr wt/-* vs wt/wt (p<0.05) | n.a. | Plasma glucose and insulin not significantly different from wt/wt; higher insulin release by pancreata undergoing glucose perfusion in *Lepr wt/-* (p<0.05) | W+, M- |
| Truett et al. 1995(102) | rat; cross between Crl (ZUC) BR and BN/Crl rats; BNZ progeny. wt/wt vs *Lepr wt/-* and *Lepr -/-* at 7 or 14 days of age | 7-day old and 14-day old *Lepr wt/-* rats showed higher body weight than wt/wt (p<0.05) | 7-day-old *Lepr wt/-* showed higher weight of inguinal adipose pads than wt/wt, but not at 14 days of age (p<0.05) | n.a. | n.a. | W+ |
| Phillips and Cleary 1994(74) | Zucker rat; wt/wt vs *Lepr wt/-* and *Lepr -/-* at 17 days of age | *Lepr wt/-* rats had lower body weight than wt/wt (p<0.05) | *Lepr wt/-* rats hat higher inguinal pad weight than wt/wt; *Lepr wt/-* rats had higher fat pad-to-body weight than wt/wt (p<0.05) | n.a. | *Lepr wt/-* rats had lower serum glucose, higher cholesterol and triacylglycerol than wt/wt (p<0.05) | W+, M+ |
| Zhang et al. 1997(78) | rat; 13M x Brown Norway Hybrids. wt/wt, *Lepr wt/-* , *Lepr -/-* at 10 days age | slightly higher in *Lepr wt/-* vs wt/wt (p<0.05) | FM and BF % significantly higher in *Lepr wt/-* vs wt/wt (p<0.05) | At comparable fat mass leptin concentration was significantly higher in *Lepr wt/-* vs wt/wt (p<0.05) | No differences in insulin concentrations between *Lepr wt/-* and wt/wt | W+, L+, M- |
| Schwarzer et al. 1997(75) | rat; 13M x Brown Norway Hybrids.wt/wt vs *Lepr wt/-* vs *Lepr -/-* at 7 and 16 days of age | wt/wt and *Lepr wt/-* were both lean | At both ages *Lepr wt/-* had significantly more BF than wt/wt (p<0.05) | n.a. | No differences in plasma insulin, glucose, TG and FFA concentrations between *Lepr wt/-* and wt/wt (isolated 2 h under cold-load) | W+, M- |
| Chung et al. 1998(41) | mouse; C57BL/6J *Lepr wt/-* and wt/wt; sacrificed between 39 and 120 days after 2-h fast | No statistically significant difference in BMI between genotypic classes of the same sex | 47.3% higher in *Lepr wt/-* vs wt/wt (p<0.05); BF 35.2% higher in *Lepr wt/-* vs wt/wt (p<0.05) | Mean levels twice as high in *Lepr wt/-* vs wt/wt; if adjusted for fat mass: leptin was 19.8% higher in *Lepr wt/-* vs wt/wt (p<0.05) | n.a. | W+, L+ |
| Kowalski et al. 1998(770) | Zucker rat; wt/wt vs *Lepr wt/-* vs *Lepr -/-* rats at postnatal day 5-18 | No significant differences in body weight among genotypes was observed at any age tested | n.a. | n.a. | n.a. | W- |
| Kraeft et al. 1999(71) | rat; offspring of Zucker rat and Brown Norway hybrid pups: wt/wt, *Lepr wt/-* and *Lepr -/-* at 1 week age | Slightly lower body weight in *Lepr wt/-* vs wt/wt (p-value not reported) | Lower fat mass in *Lepr wt/-* vs wt/wt (p<0.05) | n.a. | n.a. | W+ |
| Cleary and Phillips 1999(67) | Zucker rat; wt/wt vs female *Lepr wt/-* at 10 weeks age | Both groups lean, but body weight (p<0.05) and inguinal (p<0.05) and retroperitoneal fat (p>0.05) were heavier in *Lepr wt/-* than in wt/wt | Combined fat pad to body weight ratio higher in *Lepr wt/-* than in wt/wt (p>0.05). BF%: Sum of three fat depots (inguinal, retroperitoneal, parametrial) made a greater portion of body weight in *Lepr wt/-* vs wt/wt (p<0.05) | Serum leptin significantly higher in *Lepr wt/-* vs wt/wt (p<0.05) | No differences in serum TG, cholesterol, insulin or glucose concentrations between *Lepr wt/-* vs wt/wt | W+, L+, M- |
| Yamashita et al. 2001(64) | mice; C57BLKsJ wt/wt vs *Lepr wt/-* in pre-pregnant and pregnant state, offspring | No differences in body weight between *Lepr wt/-* and wt/wt mice in total weight; at term, *Lepr wt/-* had 33% greater weight gain than wt/wt (p<0.05) | BF%: At term, *Lepr wt/-* had 20% higher adipose tissue than wt/wt (p<0.05) | Leptin levels higher in *Lepr wt/-* mice compared with wt/wt mice (p<0.05) | Fasting glucose and insulin similar in *Lepr wt/-* and wt/wt in prepregnant state; fasting insulin increased to 3 fold in wt/wt vs to 2.2 in *Lepr wt/-* mice (p<0.05) | W+, L+, M- |
| Heo et al. 2002(68) | BNZ rat; wt/wt vs *Lepr wt/-*, after 7-week high fat vs basal diet | / | Higher epidydimal fat pad weight in *Lepr wt/-* than in wt/wt in both diets (p<0.05) | Leptin levels in homogenized adipose tissue were significantly higher in *Lepr wt/-* than in wt/wt under basal diet (p<0.05) | No difference in insulin levels between *Lepr wt/-* and wt/wt in basal diet; triglyceride levels in liver higher in *Lepr wt/-* vs wt/wt independent of diet; increased adipocyte lipogenic enzyme activity in *Lepr wt/-* vs wt/wt (p<0.05) | W+, L+, M+ |
| Tamasi et al. 2003(76) | Zucker rat; wt/wt vs *Lepr wt/-* and *Lepr -/-* at 9 and 15 weeks age | No significant differences in body weight between *Lepr wt/-* and wt/wt at any age tested | n.a. | n.a. | No significant difference in serum cholesterol, triglycerides and alkaline phosphatase between *Lepr wt/-* and wt/wt | W-, M- |
| Yamashita et al. 2003(65) | mouse; C57BLKS/J wt/wt vs *Lepr wt/-* in prepregnant and pregnant state, offspring | Prepregnant weight was slightly higher in *Lepr wt/-* than in wt/wt mice fed ad lib; maternal weight gain in *Lepr wt/-* mice fed ad lib greater than in wt/wt fed ad lib (p<0.05); offspring of *Lepr wt/-* and wt/wt similar in weight | Significantly greater body fat percentage in female offspring of *Lepr wt/-* mothers (p<0.05) | n.a. | Glucose levels similar in *Lepr wt/-* and wt/wt, while insulin higher in *Lepr wt/-* than in wt/wt in prepregnant state; higher glucose in *Lepr wt/-* than in wt/wt in pregnant state; fasting insulin higher in female offspring from *Lepr wt/-* than from wt/wt mothers (p<0.05) | W+, M+ |
| Hirose et al. 2004(56) | mouse; C57BLKS/J-wt/wt, *Lepr wt/-* and *Lepr -/-* males, at 4 weeks age | Comparable between *Lepr wt/-* and wt/wt | Epidydimal fat significantly higher in *Lepr wt/-* than in wt/wt (p<0.05) | Comparable between *Lepr wt/-* and wt/wt | Blood glucose and insulin levels comparable between *Lepr wt/-* and wt/wt | W+, L-, M- |
| Levine et al. 2006(58) | mouse; C57BL/6J wt/wt, *Lepr wt/-* and *Lepr -/-* at 12-13 weeks age | Body weight was lower in *Lepr wt/-* compared to wt/wt (p<0.05) | / | / | Blood glucose was lower in *Lepr wt/-* compared to wt/wt | W+, M+ |
| Masuyama et al. 2005(72) | rat; Spontaneously Diabetic Torii (SDT) wt/wt, *Lepr wt/-*, *Lepr -/-* at 14 weeks of age | No differences in body mass index between *Lepr wt/-* and wt/wt | Retroperitoneal and intrascapular fat pad weight similar in wt/wt and *Lepr wt/-* | No differences in leptin levels between *Lepr wt/-* and wt/wt | No differences in blood glucose and insulin levels between *Lepr wt/-* and wt/wt | W-, M- |
| Shi et al. 2007(62) | mouse; C57BL/6J wt/wt vs *Lepr wt/-* vs *Lepr -/-* at 10-12 weeks age | Slightly less body weight in *Lepr wt/-* than wt/wt (p-value not reported) | n.a. | n.a. | Blood glucose levels higher in *Lepr wt/-* than in wt/wt (p<0.05); longer reperfusion time after renal ischemia than in wt/wt (p<0.05) | W-, M+ |
| Kanda et al. 2009(57) | mouse; (obtained from Clea Japan) six-week-old male wt/wt, *Lepr wt/-* and *Lepr -/-* at 8 weeks age | Body weight of *Lepr wt/-* mice was higher than in wt/wt (p<0.05) | n.a. | n.a. | Fasting blood glucose, insulin, triglycerides higher in *Lepr wt/-* mice than in wt/wt (p<0.05); not esterified fatty acids similar in *Lepr wt/-* and in wt/wt | W+, M+ |
| Moralejo et al. 2010(73) | rat; BBDR , *Lepr wt/-* or *Lepr -/-* rat line at 30-180 days of age | No differences between wt/wt and *Lepr wt/-* | n.a. | n.a. | No differences in blood glucose between wt/wt and *Lepr wt/-* | W-, M- |
| Himeno et al. 2009(69) | Zucker rat; male wt/wt vs *Lepr wt/-* and *Lepr -/-* till age 18 weeks | No significant difference in body weight between *Lepr wt/-* and wt/wt at 18 weeks | No significant difference in epidydimal fat weight between *Lepr wt/-* and wt/wt at 18 weeks | Slightly but significantly elevated in *Lepr wt/-* vs wt/wt (p<0.05) | Slightly but significantly elevated serum insulin and triglycerid levels in *Lepr wt/-* vs wt/wt (p<0.05), serum glucose, FFA and adiponectin not significantly different between *Lepr wt/-* and wt/wt (p>0.05). Higher hepatic triglyceride levels in *Lepr wt/-* than in wt/wt; liver weight increased in *Lepr wt/-* vs wt/wt (p<0.05) | W-, L+,M+ |
| Harrod et al. 2011(55) | mouse; C57BLKS/J *Lepr wt/-* and wt/wt | *Lepr wt/-* (31 weeks old) weighed more than wt/wt (22 weeks old) (p<0.05) | n.a. | n.a. | No differences in fasting glucose  levels between wt/wt and *Lepr wt/-* mice | W+, M- |
| Stanley et al. 2011(63) | mouse; C57BL/6J wt/wt vs *Lepr wt/-* female pregnant and non-pregnant mice, offspring | No difference between non-pregnant *Lepr wt/-* and wt/wt (p>0.05); pregnant *Lepr wt/-* significantly heavier than wt/wt v; Pups born from *Lepr wt/-* mice significantly heavier than those born from wt/wt (p<0.05) | n.a. | n.a. | No difference in fasting glucose in non-pregnant *Lepr wt/-* vs wt/wt (p>0.05); fasting glucose significantly higher in pregnant *Lepr wt/-* than in wt/wt (p<0.05) | W+, M+ |
| Haldar et al. 2014(79) | sheep; SNPs identified in *Lepr* gene: chr:1:40787726; chr:1:40857869; chr:1:40858019 | Lower weight at birth and higher weight in adulthood of *Lepr wt/-* vs wt/wt (p<0.05) | n.a. | n.a. | n.a. | W+ |
| Choi et al. 2015(54) | mouse; (obtained from Korea Research Institute), wt/wt, *Lepr wt/-*, *Lepr -/-*; female 5-30 weeks age | *Lepr wt/-* and wt/wt had similar body weight | n.a. | n.a. | Blood glucose similar in *Lepr wt/-* and wt/wt | W-, M- |
| Nadif et al. 2015(59) | mouse; pregnant wt/wt, *Lepr wt/-* dams, nonpregnant counterparts, offspring | *Lepr wt/-* offspring had higher birth weight than wt/wt offspring (p<0.05);  male wt/wt offspring of *Lepr wt/-* dams had higher weight than wt/wt offspring of wt/wt dams (p<0.05) | n.a. | *Lepr wt/-* dams had higher leptin levels than wt/wt; compared to wt/wt (p<0.05), *Lepr wt/-* offspring had higher leptin levels at the age of 6 months (p<0.05) | Pregnant *Lepr wt/-* dams had impaired glucose tolerance in comparison to pregnant wt/wt (p<0.05) | W+, L+, M+ |
| Pollock et al. 2015(61) | mouse; B6.BKS(D)- *Lepr wt/-* males mated to C57Bl/6 wt/wt females to establish *Lepr wt/-* colony | *Lepr wt/-* dams body weight was significantly higher than in wt/wt dams (p<0.05); offspring from *Lepr wt/-* dams weighed significantly less than offspring of wt/wt dams at age 23-31 weeks (p<0.05) | n.a. | Leptin concentration in *Lepr wt/-* dams significantly higher than in wt/wt dams (p<0.05) | *Lepr wt/-* dams had slightly better glucose tolerance than wt/wt dam (p<0.05)s, insulin levels in *Lepr wt/-* and wt/wt were similar at gestational d. 18.5 | W+, L+, M- |
| Plows et al. 2017(60) | mouse; B6.BKS(D) and C57BL6J. *Lepr wt/-* vs wt/wt mice born from *Lepr wt/-* parents vs control | pre-pregnancy weight gain in *Lepr wt/-* mice compared to wt/wt (p<0.05) | Higher fat pads weight in *Lepr wt/-* than in wt/wt or control C57BL6J mice (p<0.05) | Hyperleptinemia in *Lepr wt/-* vs wt/wt and control mice (p<0.05) | No evidence of glucose intolerance or hyperinsulinemia in pregnant *Lepr wt/-* mice | W+, L+, M- |

Abbreviations: FFA: free fatty acids; n.a.: not available; TG: triglycerides; Differences *Lepr wt/-* vs *wt/wt*: W: weight status and body fat, L: leptin levels, M: metabolic abnormalities. -: no differences observed; +: differences observed
