# Supplementary material for: A fresh look to the phenotype in mono-allelic likely pathogenic variants of the leptin and the leptin receptor gene
Source: Mol Cell Pediatr. 2021 Aug 26;8:10. doi: 10.1186/s40348-021-00119-7 (PMC8390564; doi:10.1186/s40348-021-00119-7)
Supplement: Supplementary file 2 — Additional file 2:Table S2. Phenotype (body mass index, body fat, leptin levels and metabolic abnormalities) of human mono-allelic likely pathogenic variants of the leptin gene (LEP wt/-) in comparison to biallelic likely pathogenic variant carriers (LEP -/-), wild type controls (LEP wt/wt) and control groups. Differences between LEP wt/- and LEP wt/wt subjects were summarized in the right columns. References [123–130] details are found in Table S2 and Table S3. [file 40348_2021_119_MOESM2_ESM.docx]

**Table S2.** Phenotype (body mass index, body fat, leptin levels and metabolic abnormalities) in human mono-allelic likely pathogenic variants of the leptin gene (*LEP wt/-*) in comparison to biallelic likely pathogenic variant carriers (*LEP -/-*), wild type controls (*LEP wt/wt*) and control groups. Differences between *LEP* *wt/-* and *LEP* *wt/wt* subjects were summarized in the right columns.

| **Author, year** | ***LEP* variant**  **c.DN.A./p. position** | **Possible pathogenic consequence**  **SIFT\| PolyPhen** | **Number of carriers (children/ adults)** | **Mean BMI z-score (range) or weight status** | **Mean body fat % (range)** | **Mean leptin value ng/ml (range)** | **Metabolic abnormalities** | **Diff. between LEP wt/wt and *LEP* wt/-: Weight status (W), Leptin (L), Metab (M)** |
| --- | --- | --- | --- | --- | --- | --- | --- | --- |
| Echwald et al. 1997 (100) | n.a./p.F17L | tolerated\| benign | wt/-: n=1 (1/0) | 1.3 at age 7y, (2.9 at age 47y) | n.a. | 36.3 ng/ml at unknown age | n.a. | / |
|  | c.328G>A /p.V110M | tolerated\| benign | wt/-: n=1 (0/1) | 1.6 | n.a. | 4.5 ng/ml | n.a. | / |
| Montague et al. 1997(19) | c.398delG/ p.G133V*fs**15 | N.A.\| N.A. | wt/wt: n=3  (3/0)^a^ | NW | 17 ^c,e^ (14-20) | 6.7 ^e^  (3-12) | n.a. | W+, L- |
|  |  |  | wt/-: n=5 (1/4) | O | 33^c, e^ (15-43) | 14.2 ^e^ (3-19) | n.a. |  |
|  |  |  | -/-: n=2 (2/0) | SO | 56^c, e^ (54, 57) | 0.8 ^e^ (0.6, 1) | HI in 50% |  |
| Strobel et al. 1998(22) | c.313C>T p.R105W | deleterious\| probably damaging | wt/wt: n=1 (1/0) | n.a. | n.a. | 1.6 | no | L-, M+ |
|  |  |  | wt/-: n=8 (1/7) | 0.4 (-0.7-1.4) | n.a. | 2.6 (1.5-7.1) | HC in 43% HI in 12.5% |  |
|  |  |  | -/-: n=3 (1/2) | 4.7 (4.1-5.1) | n.a. | 1.2 (0.9-1.6) | HI in 100% |  |
| Karvonen et al. 1998(38) | c.144G>A/n.a. | N.A.\| N.A. | wt/-: n=1 (0/1) | 2.8 | 31^mn.a.^ | 4.6 | n.a. | / |
|  | c.328G>A/  p.V110M | tolerated\| benign | wt/-: n=1 (0/1) | 3.4 | 33^mn.a.^ | 3.3 | n.a. | / |
| Ozata et al. 1999(82)* | c.313C>T p.R105W | deleterious\| probably damaging | wt/wt: n=2 (0/2) | -0.2 (-1.0, 0.6) | n.a. | 2.6 (1.7, 3.5) | A in 50% | W+, L-, M+ |
|  |  |  | wt/-: n=9 (1/8) | 1.1 (0.1-2.0) | 22.6 (15-36^)i^ in n=5 | 2.6 (2.0-4.2) | HC in 25%, HTG in 37% |  |
|  |  |  | -/-: n=4 (1/3) | 4.8 (4.6-5.0) | 50.5 (43, 58)^i^ in n=2 | 1.0 (0.6-1.3) | HC in 25% HTG in 75%  A in 50% |  |
| Ozata et al. 2000(87)* | c.313C>T p.R105W | deleterious\| probably damaging | CG: n=31 (0/31) | NW and OW | n.a. | 3.8±2.4^r^ | no | L-, M- |
|  |  |  | wt/-: n=5 (1/4) | 1.1 (0.3-2.0) | n.a. | 2.2 (1.9, 2.5) | no |  |
|  |  |  | -/-: n=2 (0/2) | 4.8 (4.7, 4.9) | n.a. | 0.9 (0.6, 1.1) | HI in 100% |  |
| Ozata et al. 2001(86)* | c.313C>T p.R105W | deleterious\| probably damaging | CG: n=20 | NW and OW | n.a. | n.a. | n.a. | W- |
|  |  |  | CG: n=18 (0/18) | O | n.a. | n.a. | n.a. |  |
|  |  |  | wt/-: n=3 (1/2) | 0.3 (-0.8-0.9) | n.a. | 2.1 (1.9-2.3) | no |  |
|  |  |  | -/-: n=1 (0/1) | 4.8 | n.a. | 1.1 | HI, LGH |  |
| Farooqi et al. 2001(32) | c.398delG/ p.G133V*fs**15 | N.A.\| N.A. | CG wt/wt: n=6 | n=1 NW, n=1 OW, n=1 O | Difference in measured vs predicted BF lower than in wt/- | 27.7 ^e^ (24-32) in (n=3) | n.a. | W+, L+ |
|  |  |  | CG: n=96 (0/96) | n.a. | n.a. | 16^e^±9^r^ | n.a. |  |
|  |  |  | wt/-: n=13(1/12) | NW^e^ in n=2, OW^e^ in n=1, O^e^ in n=10 | Measured vs. predicted (41.4 vs. 34.4)^x^  (n=12) | 5.5^e^ (1-11) | n.a. |  |
| Mantzoros et al. 2001(80) | N.A. | N.A. | CG: n=5 (0/5) | NW | 17.6±4.2^r^ | 5.3 | no | W-, L+, M+ |
|  |  |  | wt/wt: n=1 | NW | n.a. | 2.8 | no |  |
|  |  |  | wt/-: n=2 (0/1, n.a. in n=1) | NW | 16.5 (14.8,18)^mn.a.^ | 0.9 (0.8-1.1) | Abn. TSH-secr. pattern |  |
|  |  |  | -/-: n=1 | O | 43 | 0.4 | Abn. TSH-secr. pattern |  |
| Lahlou et al. 2002(37)* | c.313C>T p.R105W | deleterious\| probably damaging | wt/wt: n=1 (0/1) | NW | n.a. | 2.65 | n.a. | W+, L- |
|  |  |  | CG: n=4 (0/4) | NW | n.a. | 1.5-4.5 | n.a. |  |
|  |  |  | CG: n=4 (0/4) | O | n.a. | 11.1-64 | n.a. |  |
|  |  |  | wt/-: n=13 (0/13) | 0.5  (-0.9 - 2.0) | n.a. | 1.9 (1.0-4.5) | n.a. |  |
|  |  |  | -/-: n=4 (1/3) | 5.1 (4.5-6.0) | n.a. | 0.6 (0.5-0.6) | n.a. |  |
| Paz-Filho et al. 2008(121) | c.313C>T p.R105W | deleterious\| probably damaging | wt/-: n=3 (0/2, n.a. in n=1) | n.a. | n.a. | n.a. | n.a. | / |
|  |  |  | -/-: n=1 (1/0) | 3.5 | n.a. | n.a. | HI, HTG |  |
| Mazen et al. 2009(123) | c.309C>A p.N103K | deleterious\| probably damaging | wt/-: n=2 (0/2) | 1.5 (1.4, 1.7) | n.a. | n.a. | n.a. | / |
|  |  |  | -/-: n=2 (2/0) | 9.0 (5.5, 12.5) | n.a. | 1.2 (1.1, 1.3) | HI in 100% |  |
| Fischer-Posovszky et al. 2010(124) | c.215T>C  p.L72S | deleterious\| probably damaging | wt/-: n=9 (0/6, age n.a. in n=3) | 0.9 (0.5-1.5) in n=6 | n.a. | 2.6 (0.7-5.1) in n=6 | n.a. | / |
|  |  |  | -/-: n=1 (1/0) | 2.7 | 50.1^x^ | 0.4 | HI, DL |  |
| Fatima et al. 2011(90) | c.398delG p.G133V*fs**15 | N.A.\| N.A. | wt/-: n=12 (0/12) | n.a. | n.a. | n.a. | n.a. | / |
|  |  |  | -/-: n=7 (7/0) | 5.1 (3.2-6.9) | n.a. | 0.3 (0.1, 0.5) in n=2 | n.a. |  |
|  | c.104_106delTCA/  p.I35del/N-termin.a.l domain | N.A.\| N.A. | CG: n=2 (2/0) | BMI-matched to hom | n.a. | 87.7 (42.3, 133.1) | n.a. | / |
|  |  |  | wt/-: n=3 (0/2, n.a. in n=1) | NW | n.a. | n.a. | n.a. |  |
|  |  |  | -/-: n=1 (1/0) | 6.5 | n.a. | 3.6 | n.a. |  |
|  | c.481_482delCT/  p.L161G*fs**170 | N.A.\| N.A. | CG: n=2 (2/0) | BMI-matched to -/- | n.a. | 32.0 (14.1, 50.0) | n.a. | / |
|  |  |  | wt/-: n=2  (0/2) | n.a. | n.a. | n.a. | n.a. |  |
|  |  |  | -/-: n=1 (1/0) | 4.7 | n.a. | 0.2 | n.a. |  |
| Murray et al. 2011(81) | c.68C>G/  p.P23R | deleterious\| probably damaging | wt/wt: n=1 (0/1) | OW | n.a. | n.a. | n.a. | W+ |
|  |  |  | wt/-: n=3 (1/1,age n.a. in n=1) | -2.4 (n=1), thin (n=1), NW (n=1) | n.a. | 1.0 in n=1 | no |  |
| Saeed et al. 2012(86) | c.104_106delTCA/  p.I35del | N.A.\| N.A. | CG: n=20 (20/0) | NW | n.a. | 4.0±0.6^r^ | no | L+ |
|  |  |  | wt/-: n=3 (1/2) | 0.0  (-0.8 - 0.4) | n.a. | 8.8 (1.0- 24.0) | HI in 33% |  |
|  |  |  | -/-: n=1 (1/0) | 5.5 | n.a. | <0.2 | High cortisol |  |
| Thakur et al. 2014(125) | c.163C>T/  p.Q55X | N.a.\| N.A. | wt/-: n=2 (0/2) | n.a. | n.a. | n.a. | n.a. | / |
|  |  |  | -/-: n=1 (1/0) | 5.2 | n.a. | <0.6 | HI in 100% |  |
| Saeed et al.2 014(88) | c.398delG p.G133V*fs**15 | N.A.\| N.A. | CG wt/wt: n=10 (0/10) | NW | n.a. | 3.5 (1-7)^e^ | no | W+, L+, M- |
|  |  |  | wt/-: n=8 (0/8) | 1.2  (-0.1 - 2.4) | n.a. | 6.9 (2-13)^e^ | no |  |
|  |  |  | -/-: n=5 | O | n.a. | nd | HI |  |
| Zhao et al. 2014(89) | c.353A>T/  p.H118L | deleterious\| benign | wt/-: n=1 (0/1) | 4.2 | n.a. | nd | HT, MS, HS | / |
| Saeed et al. 2015(95) | c.1-44del42/  n.a. | N.A.\| N.A. | wt/-: n=2 (0/2) | n.a. | n.a. | n.a. | n.a. | / |
|  |  |  | -/-: n=1 (1/0) | 4.3 | n.a. | nd | HI |  |
|  | c.350G>A/  p.C117Y | deleterious\| probably damaging | wt/-: n=2 (0/2) | n.a. | n.a. | n.a. | n.a. | / |
|  |  |  | -/-: n=1 (1/0) | 4.1 | n.a. | nd | no |  |
| Shaban.a. and Hasn.a.in 2016(33) | c.309C>A p.N103K | deleterious\| probably damaging | wt/wt: n=2 | NW | n.a. | n.a. | n.a. | W+ |
|  |  |  | wt/-: n=3 (0/3) | 1.2 (0.7-1.5) | n.a. | n.a. | n.a. |  |
|  |  |  | -/-: n=1 (1/0) | 4.6 | n.a. | 0.9 | n.a. |  |
| Wabitsch et al. 2017(35) | c.298G>T/  p.D100Y | deleterious\| probably damaging | wt/-: n=2 (0/2) | 1.2 (1.1-1.3) | n.a. | 3.2 (1.1, 5.3)^b^ | n.a. | / |
|  |  |  | -/-: n=1 (1/0) | 9.8 | 53.4^x^ | nd^b^ | n.a. |  |
|  | c.309C>A p.N103K | deleterious\| probably damaging | wt/-: n=2 (0/2) | 0.9 (0.5, 1.2) | n.a. | 3.0 (1.7, 4.3)^b^ | n.a. | / |
|  |  |  | -/-: n=2 (2/0) | 5.4 (4.2, 6.5) | 52.1 (51.8, 52.3)^x^ | nd^b^ | n.a. |  |
| Nordang et al. 2017(96) | c.280G>A/  p.V94M | tolerated\| benign | wt/-: n=1 (0/1) | O | n.a. | n.a. | n.a. | / |
|  | c.53A>G/  p.Y18C | tolerated\| benign | wt/-: n=4 (0/4) | O (n=3), L (n=1) | n.a. | n.a. | n.a. | / |
| Dayal et al. 2018(126) | c.298G>A/  p.D100N § | deleterious\| probably damaging | wt/-: n=2 (0/2) | NW | n.a. | n.a. | n.a. | / |
|  |  |  | -/-: n=1 (1/0) | 8.2 | n.a. | 1.3 | HTh |  |
| Yupanqui-Lozno et al. 2019(83) | c.350G>T/  p.C117F | deleterious\| benign | wt/wt: n=2 (1/1) | 0.6 (0.4, 0.8) | n.a. | n.a. | n.a. | W+ |
|  |  |  | wt/-: n=2 (0/2) | 1.4 (0.6, 2.1) | n.a. | n.a. | n.a. |  |
|  |  |  | -/-: n=2 (2/0) | 4.3 (3.5, 5.1) | n.a. | nd | HTG, IR in 100% |  |

**Abbreviations**: A: anemia; CG: not related control group; CG WT: not related wt/wt control group; diff: difference; DL: dyslipidemia, HC: hypercholesterinemia>200 mg/dl; wt/-: mono-allelic likely pathogenic variant; HI: hyperinsulinemia; -/-: biallelic-pathogenic variant; HS: hepatic steatosis; HT: hypertension; HTG: hypertriglyceridemia; Hth: hypothyreosis; IR: insulin resistance; LGH: low growth hormone; MS: metabolic syndrome; Metab: metabolic abnormalities; NW: normal weight; n.a.: not available; nd: not detectable; no: no abnormalities found among reported parameters; O: obese; OW: overweight; SO: severely obese. Differences between *LEP wt/-* vs. *LEP wt/wt*: W weight status and body fat, L:leptin levels, M:metabolic abnormalities; -: no differences observed, +: differences observed. Difference in body mass was reported if BMI z-score> 1 in one category and not in the other, or if range was not overlapping; /: no comparison possible. ^a^ subjects categorized as adults; ^c^: calculated body fat as reported in study; **^e^**: value extracted from figure; ^i^: body fat measured by electric impedance; ^L^: as reported in cited study; ^mn.a.^: method of body fat measurement not available; ^PP2^: as predicted by Polyphen-2 software; ^x^: body fat measured by x-ray absorptiometry;  ^r^: values as reported in study (no range available); *double reporting cannot be excluded; § Heterozygous and homozygous *LEP* mutation carriers carried addition.a.lly a *BBS1* mutation.
