# Supplementary material for: A fresh look to the phenotype in mono-allelic likely pathogenic variants of the leptin and the leptin receptor gene
Source: Mol Cell Pediatr. 2021 Aug 26;8:10. doi: 10.1186/s40348-021-00119-7 (PMC8390564; doi:10.1186/s40348-021-00119-7)
Supplement: Supplementary file 3 — Additional file 3:Table S3. Phenotype (body mass index, body fat, leptin levels and metabolic abnormalities) of human mono-allelic likely pathogenic variants of the leptin receptor gene (LEPR wt/-) in comparison to biallelic likely pathogenic variant carriers (LEPR -/-), wild type controls (LEPR wt/wt) and control groups. Differences between LEPR wt/- and LEPR wt/wt subjects were summarized in the right columns. References [123–130] details are found in Table S2 and Table S3. [file 40348_2021_119_MOESM3_ESM.docx]

**Table S3.** Phenotype (body mass index, body fat, leptin levels and metabolic abnormalities) in human mono-allelic likely pathogenic variants of the leptin receptor gene (*LEPR wt/-)* in comparison to biallelic likely pathogenic variant carriers (*LEPR -/-*), wild type controls (*LEPR wt/wt*) and control groups. Differences between *LEPR wt/-* and *LEPR wt/wt* subjects were summarized in the right columns.

| Ref. | ***LEPR* variant**  **c.DNA/p. position** | ***Nomenclature (HGVS)*** | **Possible pathogenic consequence**  **SIFT\| PolyPhen** | **Number of carriers (children/ adults)** | **Mean BMI z-score:**  **(range)**  **or weight status** | **Mean body fat % (range)** | **Mean leptin value ng/ml (range)** | **Metabolic abnormalities** | **Differences between LEPR wt/wt and *LEPR* wt/-: Weight status (W), Leptin (L), Metab (M)** |
| --- | --- | --- | --- | --- | --- | --- | --- | --- | --- |
| Clement et al. 1998 (20) | G>A in splice donor site of exon 16/na | c.2597+1G>A | n.a.\| n.a. | wt/wt: n=2 (2/0) | 0.9 (-0.9, 2.7) | 49^ba^ in n=1 | 46.8 (5.6, 88) | HI in 50% | W-, L+, M+ |
|  |  |  |  | wt/-: n=6 (2/4) | 1.8 (1.3-2.4) in n=5 | 32^ba^ (20-42) in n=3 | 250.6 (145-362) in n=5 | HC in 33.3%, HTG in 16.7% |  |
|  |  |  |  | -/-: n=3 (1/2) | 4.8 (4.5-5.2) | 67^ba^ (66, 68 in n=2) | 598.7 (526-670) | HI in 33.3% |  |
| Lahlou et al. 2000* (36) | G>A in splice donor site of exon 16/na | c.2597+1G>A | n.a.\| n.a. | wt/wt: n=2 (2/0) | 1.0  (-0.7 - 2.7) | 49^ba^ in n=1 | 72.2^f^ in n=1 | n.a. | W-, L- |
|  |  |  |  | CG: n=10 (0/10) | O | n.a. | 91±19 ^f^ | n.a. |  |
|  |  |  |  | wt/-: n=5  (1/4) | 1.8 (1.1-2.6) | 32^ba^ (19.5-42) in n=3 | 35.4 ^f^ (3.5-52) in n=4 | n.a. |  |
|  |  |  |  | -/-: n=3 (2/1) | 4.7 (4.1-5.3) | 67^ba^ (66, 68) in n=2 | 114 ^f^(92-139) | n.a. |  |
| Lahlou et al. 2002* (37) | G>A in splice donor site of exon 16/na | c.2597+1G>A | n.a.\| n.a.\| | wt/wt: n=2 (2/0) | 1.0 (-0.7, 2.7) | n.a. | 72.2^f^ in n=1 | n.a. | W-, L- |
|  |  |  |  | CG: n=10 (0/10) | O | n.a. | 91±19 ^f^ | n.a. |  |
|  |  |  |  | wt/-: n=5 (1/4) | 1.8 (1.1-2.6) | n.a. | 35.4 ^f^  (3.5-52) in n=4 | n.a. |  |
|  |  |  |  | -/-: n=3 (2/1) | 4.7 (4.1-5.3) | n.a. | 104 ^f^(92-109) | n.a. |  |
| Branson R. et al. 2003 (127) Genebank Ref. Sequence: AC097063.2 | G97244A/  p.R612H | c.1835G>A  p.R612H | tolerated\| probably damaging | wt/-: n=1 | SO | n.a. | n.a. | n.a. | / |
|  | T97307A/  p.V633N | c.1898T>A  p.V633N | delirious\| probably damaging | wt/-: n=1 | SO | n.a. | n.a. | n.a. |  |
| Farooqi I. et al. 2007 (91) | 4 bp deletion in codon 22/na | Del(4bp) Codon22 | n.a.\| n.a. | wt/wt: n=2 (2/0) | 1.0 (0.2, 1.8) | 30^x^ (18, 42) | n.a. | n.a. | W- |
|  |  |  |  | wt/-: n= 5 (1/4) | 0.7 (0.1-1.4) | 32^x^ (21-38) | n.a. | n.a. |  |
|  |  |  |  | -/-: n=3 (3/0) | 4.8 (3.3-7.6) | 43^x^ (42, 44) in n=2 | 103.5 (97,110) in n=2 | HI in 66,7% |  |
|  | 11-bp deletion in codon 70/na | Del(11bp) Codon70 | n.a.\| n.a. | wt/-: n=2 (0/2) | 1.2 (1.0, 1.4) | 37 (27, 47) | n.a. | n.a. | / |
|  |  |  |  | -/-: n=2 (2/0) | 4.4 (4.0, 4.7) | 58 ^x^  (58, 58) | 271.5 (178, 365) | HI in 100% |  |
|  | 66 bp deletion in codon 514/na | Del(66bp) Codon514 | n.a.\| n.a. | wt/-: n=2 (0/2) | 0.8 (0.3, 1.2) | n.a. | n.a. | n.a. | / |
|  |  |  |  | -/-: n=1 (1/0) | 10 | n.a. | n.a. | n.a. |  |
|  | na/p.W31X | p.W31* | n.a.\| n.a. | wt/-: n=4 (0/4) | 0.2  (-0.9 - 1.2) | n.a. | n.a. | n.a. | / |
|  |  |  |  | -/-: n=3 (0/3) | 5.1 (4.2-6.1) | n.a. | 134.3 (90-180) | DM in 66.7% |  |
|  | p.A409E | c.1226C>A  p.A409E | deleterious\| probably damaging | wt/-: n=2 (0/2) | 1.3 (1.0, 1.6) | n.a. | n.a. | n.a. | / |
|  |  |  |  | -/-: n=1 (1/0) | 9.2 | n.a. | 36 | no |  |
|  | p.W664R |  | deleterious\| probably damaging | wt/-: n=2 (0/2) | 1.6 (1.5, 1.6) | 41^x^  (40, 41) | n.a. | n.a. | / |
|  |  |  |  | -/-: n=1 (1/0) | 4.9 | 60 ^x^ | 194 | no |  |
|  | p.H684P | c.2051A>C  p.H684P | tolerated\| benign | wt/wt: n=2 (1/1) | 0.5 (-0.6, 1.6) | 20 ^x^  (19, 21) | n.a. | n.a. | W- |
|  |  |  |  | wt/-: n=2 (0/2) | 0.5 (-0.1, 1.1) | 21^x^  (18, 24) | n.a. | n.a. |  |
|  |  |  |  | -/-: n=1 (1/0): | 4.2 | 47 ^x^ | 14 | no |  |
|  | 1 bp deletion in codon 15/na (V1) **and** na/ p.R612H (V2) | Del(1bp) Codon15 | V1: NA\| NA  V2: reduced signalling^F^ | wt/wt: n=2 (0/2) | 0.3 (-0.6, 1.2) | 20 ^x^  (18, 22) | n.a. | n.a. | W- (V1) W+ (V2) |
|  |  | c.1835G>A  p.R612H |  | wt/- for V1: n=2 (1/1) | 0.1 (-1.6, 1.7) | 32 ^x^  (27, 44) | n.a. | n.a. |  |
|  |  |  |  | wt/- for V2: n=1 (0/1) | 2.4 | 27 ^x^ | n.a. | n.a. |  |
|  |  |  |  | Comp. het for V1 and V2: n=1 (1/0) | 3.6 | 41 ^x^ | n.a. | n.a. |  |
| Mazen et al. 2011 (128) | C-A transition exon 6/p.P316T | c.946C>A  p.P316T | deleterious\| benign | wt/-: n=4 (0/4) | n.a. | n.a. | n.a. | n.a. | / |
|  |  |  |  | -/-: n=2 (2/0) | 6.8 (5.6, 7.9) | n.a. | 46 (40, 52) | HT in 100% |  |
|  |  |  |  | -/- for V1 and V2: n=1 (0/1) | 6.7 | n.a. | 100 | HI in 100% |  |
| Saeed et al. 2014b (94) | c.2396-1G>T/  p.799-1G>T | c.2396-1G>T | n.a.\| n.a. | wt/wt: n=2 (1/1) | 1.9 (1.1, 2.7) | n.a. | n.a. | n.a. | W- |
|  |  |  |  | wt/-: n=6 (0/6) | 0.1  (-0.9 - 1.4) | n.a. | 3.3 (2.2, 4.4) in n=2 | no |  |
|  |  |  |  | -/-: n=1 (1/0) | 6.8 | n.a. | 76.8 | HI, HCo |  |
|  | c.1675 G>A/ p.W558* | c.1674 G>A  p.W558* | n.a.\| n.a. | wt/-: n=2 (0/2) | 1.9 (1.7, 2.1) | n.a. | 28.0 (10.8, 45.1) | no | / |
|  |  |  |  | -/-: n=1 (1/0) | 7.9 | n.a. | 71.7 | no |  |
| Saeed et al. 2015 (95) | c.1810T>A/ p.C604S | c.1810T>A  p.C604S | deleterious\| probably damaging | wt/-: n=2  (0/1, na in n=1) | NW in n=1 | n.a. | n.a. | n.a. | M- |
|  |  |  |  | -/-: n=2 (2/0) | SO | n.a. | n.a. | n.a. |  |
|  | c.2396-1G>T/na | c.2396-1G>T | n.a.\| n.a. | wt/-: n=6 (0/6) | n.a. | n.a. | n.a. | n.a. | / |
|  |  |  |  | -/-: n=3 (3/0) | SO | n.a. | n.a. | na |  |
|  | c.1675G>A/ p.W558* | c.1674 G>A  p.W558* | n.a.\| n.a. | wt/-: n=2 (0/2)= | n.a. | n.a. | n.a. | n.a. | / |
|  |  |  |  | -/-: n=1 (1/0) | SO | n.a. | n.a. | n.a. |  |
| Huvenne et al. 2015 (93) | c.1810T>G/ p.C604G | c.1810T>G  p.C604G | deleterious\| probably damaging | wt/-: n=2 (0/2) | n.a. | n.a. | n.a. | n.a. | / |
|  |  |  |  | -/-: n=1 (0/1) | 5.0 | 56.6^x^ | 136.1 | HC, IR |  |
|  | c.2357T>C/ p.L786P | c.2357T>C  p.L786P | deleterious\| probably damaging | wt/-: n=4 (1/3) | 1.4 (1.3, 1.6) in n=2, SO in n=1 | n.a. | n.a. | n.a. | / |
|  |  |  |  | -/-: n=1 (1/0) | 4.0 | 36.3 ^x^ | 162.4 | no |  |
|  | c.2491G>A/ p.H800-N831del | c.2491G>A | tolerated\| benign | wt/-: n=2 (0/2) | 0.7 (0.6, 0.7) | n.a. | n.a. | n.a. | / |
|  |  |  |  | -/-: n=1 (1/0) | 8.6 | 65 ^x^ | 36.4 | no |  |
|  | c.δexon6-8/p.P166CfsX7 (V1) **and** c.1604-1G>A/ p.535-1G>A (V2) | c.(494+1_495-1)_(994+1_995-1)del  p.(P166Cfs*) | -  n.a.\| n.a. | wt/wt: n=6 (0/6) | 2.8 (0.4-5.0) | 41^x^ (20- 52) | n.a. | HC in 50% in n=6 | W-, M+ |
|  |  | c.1604-1G>A |  | wt/- for V1: n=8 (0/8) | 2.0 (-0.6-3.7) in n=6 | 43^x^ (38.6-48.2) in n=4 | n.a. | HC and HTG in 66,7% in n=3 |  |
|  |  |  |  | Comp het for V1 and V2: n=1 (1/0) | 3.7 | n.a. | n.a. | n.a. |  |
|  |  |  |  | -/- for V1: n=5 (3/2) | 5.7 (3.8-10.6) | 52^x^ (49-54.7) in n=3 | 81.8 (53-100) | IR in 20%, HC in 20% |  |
|  | c.1264T>C/ p.Y422H (V1) **and** c.2131dup/ p.T711NfsX18 (V2) | c.1264T>C  p.Y422H | V1: deleterious\| probably damaging  V2: n.a.\| n.a. | wt/wt: n=3 (0/3) | 1.8 (1.1, 2.5) in n=2 | n.a. | n.a. | n.a. | W- |
|  |  | c.2131dupA p.T711Nfs*18 |  | wt/- V1: n=3 (0/3) | 0.1  (-1.0 - 0.8) | n.a. | n.a. | n.a. |  |
|  |  |  |  | wt/- V2: n=5 (0/5) | 1.5 (0.8-2.8) in n=4 | n.a. | n.a. | n.a. |  |
|  |  |  |  | Comp wt/-: n=2 (0/2) | 4.3 (4.3, 4.3) | 10.3 ^x^ (4.4, 16.2) | 51 (50.7, 51.8) | TG in 50%, IR in 50% |  |
| Hannema et al. 2016 (92) | c.1753-1dupG intron 13/ p.M585Dfs*2 (V1)  **and** c.2168C>T exon 16/ p.S723F (V2) | c.1753-1dupG | V1: n.a.\| n.a. V2: deleterious\| probably damaging | wt/wt: n=1 (0/1) | 1.3 | n.a. | n.a. | n.a. | W+ (V1) W- (V2) |
|  |  |  |  | wt/- for V1: n=1 (0/1) | 3.3 | n.a. | n.a. | n.a. |  |
|  |  | c.2168C>T  p.S723F |  | wt/- for V2: n=2 (1/1) | 0.0 (-0.1, 0.2) | n.a. | n.a. | n.a. |  |
|  |  |  |  | Comp wt/-: n= 1 (1/0) | 3.8 | 43.7^x^ | 67.2 | HI, HT in 100% |  |
| Nordang et al. 2017 (96) | c.96A>T/ p.R32S |  | tolerated\| benign | wt/-: n=1 (0/1) | SO | n.a. | n.a. | n.a. | / |
|  | c.1178T>C/ p.F393S | c.1178T>C  p.F393S | deleterious\| possibly damaging | wt/-: n=1 (0/1) | 3.8 | n.a. | n.a. | DM 2, HT, MS |  |
|  | c.1246C>T/ p.H416Y | c.1246C>T  p.H416Y | tolerated\| possibly damaging | wt/-: n=1 (0/1) | SO | n.a. | n.a. | n.a. |  |
|  | c.1813G>T/ p.A605S | c.1813G>T  p.A605S | deleterious\| benign | wt/-: n=1 (0/1) | SO | n.a. | n.a. | n.a. |  |
|  | c.2260G>A/ p.V754M | c.2260G>A  p.V754M | deleterious\| probably damaging | wt/-: n=1 (0/1) | SO | n.a. | n.a. | n.a. |  |
|  | c.3320C>G/ p.P1107R | c.3320C>G  p.P1107R | tolerated\| benign | wt/-: n=1 (0/1) | SO | n.a. | n.a. | n.a. |  |
|  | c.3493_3494insC/ p.V1165fs |  | n.a.\| n.a. | wt/-: n=1 (0/1) | SO | n.a. | n.a. | n.a. |  |
|  | c.371-8A>T/ na | c.371-8A>T | n.a.\| n.a. | wt/-: n=3 (0/3) | NW in n=1, SO in n=2 | n.a. | n.a. | n.a. |  |
|  | c.2208C>G/ p.S736R | c.2208C>G  p.S736R | deleterious\| probably damaging | wt/-: n=1 (0/1) | NW | n.a. | n.a. | n.a. |  |
|  | c.2362A>C/ p.I788L | c.2362A>C  p.I788L | deleterious\|benign | wt/-: n=1 (0/1) | NW | n.a. | n.a. | n.a. |  |
| Dehghani et al. 2018 (129) Transcript NM 601007 | c.464T>G p.Y155* | c.465T>G  p.Y155* | n.a. | wt/-: n=3 (1/2) | na | n.a. | n.a. | n.a. | / |
|  |  |  |  | -/-: n=9 | 4.6 (0.4-7.6) in n=8 | n.a. | n.a. | n.a. |  |
| Akinci et al. 2019 (130) | c.12A>C  p.Q4H | c.12A>C  p.Q4H | deleterious\| benign | wt/-: n=1  (1/1) | 3.4 in n=1  2.6 in n=1 | n.a. | n.a. | n.a. | / |
| Voigtmann et al. 2021 (97) | p.Arg612His | c.1835G > A p.R612H | tolerated\| probably damaging | wt/-: n=1  (1/1) | 3.73 in n=1  0.21 in n=1 | n.a. | 10.8 | n.a. | / |

**Abbreviations**: DM 2: diabetes mellitus 2; HC: hypercholesterinemia; wt/-: mono-allelic likely pathogenic variant; HCo high cortisol values; HG: hyperglycemia; HI: hyperinsulinemia; -/-: biallelic-pathogenic variant; HTG: hypertriglyceridemia; HT: hypertension; IR: insulin resistance; MS: metabolic syndrome; n.a.: not available; no: no abnormalities observed within reported parameters; O: obese; OW: overweight; SO: severely obese; V1: variant 1; V2: variant 2. Differences *LEPR wt/-* vs *LEPR wt/wt*: W weight status and body fat, L: leptin levels, M: metabolic abnormalities; -: no differences observed, +: differences observed. Difference in body mass was reported if BMI z-score> 1 in one category and not in the other, or if range was not overlapping; /: no comparison possible. ba: biphotonic absorptiometry measurement of fat mass; f free leptin as reported in the study; F: functional study performed for analysis of variants; L: pathogenic mechanism as reported in cited study; P: predicted pathogenic consequence; PP2: prediction by Polyphen-2 prediction tool r: values as reported, no range available; x: dual energy x-ray absorptiometry scanning for measurement of fat mass
